# Systemic Metabolomics in a Framework of Genetics and Lifestyle in Age-Related Macular Degeneration

**DOI:** 10.3390/metabo13060701

**Published:** 2023-05-27

**Authors:** Eric F. Thee, İlhan E. Acar, Johanna M. Colijn, Magda A. Meester-Smoor, Timo Verzijden, Sara J. Baart, Mohamed A. Jarboui, Sascha Fauser, Carel B. Hoyng, Marius Ueffing, Anneke I. den Hollander, Caroline C. W. Klaver

**Affiliations:** 1 Department of Ophthalmology, Erasmus University Medical Center, 3015 GD Rotterdam, The Netherlands; e.thee@erasmusmc.nl (E.F.T.); j.m.colijn@erasmusmc.nl (J.M.C.); m.meester-smoor@erasmusmc.nl (M.A.M.-S.); 2 Department of Epidemiology, Erasmus University Medical Center, 3015 GD Rotterdam, The Netherlands; t.verzijden@erasmusmc.nl (T.V.); s.baart@erasmusmc.nl (S.J.B.); 3 Department of Ophthalmology, Donders Institute for Brain, Cognition and Behaviour, Radboud University Medical Center, 6525 GA Nijmegen, The Netherlands; erkin.acar@bsse.ethz.ch (İ.E.A.); carel.hoyng@radboudumc.nl (C.B.H.); 4 Department of Biostatistics, Erasmus University Medical Center, 3015 GD Rotterdam, The Netherlands; 5 Department of Ophthalmology, Institute for Ophthalmic Research, Eberhard Karls University of Tübingen, 72076 Tübingen, Germany; mohamed-ali.jarboui@uni-tuebingen.de (M.A.J.); marius.ueffing@uni-tuebingen.de (M.U.); 6 Department of Ophthalmology, University Eye Clinic, Eberhard Karls University of Tübingen, 72076 Tübingen, Germany; 7 Department of Ophthalmology, University Hospital Cologne, 50937 Cologne, Germany; sascha.fauser@gmail.com; 8 Hoffman-La Roche AG, 4070 Basel, Switzerland; 9 AbbVie, Genomics Research center, Cambridge, MA 02139, USA; anneke.denhollander@abbvie.com; 10 Institute of Molecular and Clinical Ophthalmology, University of Basel, 4070 Basel, Switzerland

**Keywords:** age-related macular degeneration, Europe, metabolomics, genetics, lifestyle

## Abstract

Insights into the pathogenesis of age-related macular degeneration (AMD), a leading cause of blindness, point towards a complex interplay of genetic and lifestyle factors triggering various systemic pathways. This study aimed to characterize metabolomic profiles for AMD and to evaluate their position in the trias with genetics and lifestyle. This study included 5923 individuals from five European studies. Blood metabolomics were assessed using a nuclear magnetic resonance platform of 146 metabolites. Associations were studied using regression analyses. A genetic risk score (GRS) was calculated using β-values of 49 AMD variants, a lifestyle risk score (LRS) using smoking and diet data, and a metabolite risk score (MRS) using metabolite values. We identified 61 metabolites associated with early-intermediate AMD, of which 94% were lipid-related, with higher levels of HDL-subparticles and apolipoprotein-A1, and lower levels of VLDL-subparticles, triglycerides, and fatty acids (false discovery rate (FDR) *p*-value < 1.4 × 10^−2^). Late AMD was associated with lower levels of the amino acids histidine, leucine, valine, tyrosine, and phenylalanine, and higher levels of the ketone bodies acetoacetate and 3-hydroxybutyrate (FDR *p*-value < 1.5 × 10^−3^). A favorable lifestyle characterized by a healthy diet was associated with higher levels of amino acids and lower levels of ketone bodies, while an unfavorable lifestyle, including smoking, showed opposite effects (FDR *p*-value < 2.7 × 10^−2^). The MRS mediated 5% of the effect of the GRS and 20% of that of the LRS on late AMD. Our findings show that metabolomic profiles differ between AMD stages and show that blood metabolites mostly reflect lifestyle. The severity-specific profiles spur further interest into the systemic effects related to disease conversion.

## 1. Introduction

Age-related macular degeneration (AMD) is the most common cause of blindness among the elderly in European countries. The disease primarily affects the central part of the retina that provides the sharpest visual perception. AMD is initially characterized by early and intermediate stages in which yellow deposits (drusen) form under the retinal pigment epithelium and retinal pigmentary changes develop; usually without much notice. The initial stages may progress to the loss of retinal pigment epithelium and photoreceptors (dry AMD) or cause neovascularization (wet AMD). The latter may benefit from anti-vascular endothelial growth factor therapy, but converts to retinal degeneration at longer term. The number of blind individuals due to AMD was estimated at approximately 10 million in 2020 and is projected to double by 2040 [1,2]. Recent insights into the disease etiology point towards a complex interplay of genetic and lifestyle factors; 52 common risk variants in 34 loci determine the prominent genetic susceptibility and lifestyle factors, including smoking and diet, are known to increase the risk of AMD considerably. These risk factors are known to trigger prominent systemic as well as local pathways, such as the complement cascade, lipid metabolism, extracellular matrix remodeling, and oxidative stress [3,4,5,6,7,8,9,10]. Established determinants that can be measured in blood include complement activation markers and HDL, but it is likely that more systemic factors are at play. This calls for more in-depth insight into the systems biology of AMD.

Metabolomics is a recent area of interest that investigates small molecules related to cellular metabolism [11,12,13,14,15,16,17,18]. Metabolomic studies on the systemic level are attractive as blood metabolites directly reflect genetic, behavior, and lifestyle factors, and are known to cross the blood–retina barrier [19]. Moreover, systemic metabolites may have clinical application as blood is easily accessible [19]. Over the years, many studies have investigated single or a small number of systemic markers, but only a few studies used comprehensive metabolomic platforms [20]. In a previous study, we identified over 60 blood metabolites associated with AMD with a nuclear magnetic resonance (NMR)-based array [11,21]. However, it is still unclear how these metabolites reflect genetic and lifestyle factors, and whether they are biomarkers for a certain stage of disease.

In this study, we aimed to bring metabolomics in AMD to the next level of knowledge by characterizing metabolomic profiles for the stages of AMD and by evaluating the position of metabolomics in the trias with genetics and lifestyle.

## 2. Materials and Methods

### 2.1. Study Population

Three population-based studies from The Netherlands (Rotterdam Study, [RS]), and two case control studies, i.e., one from Germany (European Genetic Database [EUGENDA-Cologne]), and one from the Netherlands (EUGENDA-Nijmegen), were included in the study. All studies participated in the European Eye Epidemiology consortium and EYE-RISK project. A detailed description of the consortium was published previously [22]. Participating studies were conducted according to the tenets of the Declaration of Helsinki, and written informed consent was obtained from all participants. The recruitment and research protocols were reviewed and approved by the local institutional review boards; the EUGENDA study was approved by the ethics committees in Cologne and Nijmegen; the Rotterdam Studies I, II, and III were approved by the Medical Ethics Committee of the Erasmus MC (registration number: MEC 02.1015); and by the Dutch Ministry of Health, Welfare and Sport. A total of 5923 individuals had gradable fundus photographs and metabolomics information. Of those, 3836 had data on genetics and lifestyle factors additionally available. For incidence and progression analyses, we excluded individuals who already had reached investigated endpoints at baseline (early-intermediate AMD, late AMD) and had no follow-up (Figure S1).

### 2.2. Evaluation of Metabolites

EDTA plasma samples of 5923 individuals were analyzed on a nuclear magnetic resonance (NMR)-based platform (Nightingale Health Ltd., Helsinki, Finland) for metabolomics measurements [21]. This platform provided 225 measurements from the plasma samples (full list available at: https://nightingalehealth.com/biomarkers (accessed on 21 June 2021)). Among these 225 measurements, there were 79 derivative results, i.e., ratios, percentages, or sums of already available individual metabolite levels. These derivative values were removed from the data set, leaving 146 metabolite levels. Samples from EUGENDA-Nijmegen and EUGENDA-Cologne studies were shipped and measured in one batch on the Nightingale metabolomics platform version 2016. RS study samples were previously sent in a different batch and measured for metabolite levels on the 2014 version. β coefficients for metabolite associations with late AMD were calculated using a univariate logistic regression. Metabolites associated with late AMD (8 in total) were multiplied by determinant values and were summed to create a metabolomic risk score (MRS).

### 2.3. Evaluation of the Genetic and Lifestyle Determinants

The RS participants were genotyped using Illumina 550 K, 550 K due/610 K Illumina arrays (Illumina, Inc., San Diego, CA, USA) and imputed with the 1000 Genomes (phase 1 version 3) reference panel using Markov chain haplotyping/minimac software [6]. EUGENDA participants were genotyped with single-molecule molecular inversion probes (smMIPs) in combination with next generation sequencing (NGS), or exome chip analysis, as described previously [7,23]. All studies applied similar quality control procedures to genotype data before analysis; imputation quality was r2 > 0.3.

Genetic risk scores (GRSs) for 49 AMD-associated risk variants (total GRS) and for different biological pathways (ARMS2/HTRA1, complement, lipid, extracellular matrix) were calculated as the sum of the βs of the risk variants from the GWAS of the International AMD Genomics Consortium, as described previously [6,7,24].

Four well-established AMD lifestyle determinants (smoking status and servings of vegetables, fruit, and fish per day) were assessed using a questionnaire. Smoking status was categorized as no, former, or current smoker. Dietary intake of vegetables, fruit, and fish, was analyzed in medium servings per day with a maximum of 120 g of vegetables per day, 120 g of fruit per day, and 100 g of fish per day. Lifestyle risk scores (LRSs) were calculated as the sum of the βs of smoking status and dietary intakes, as described previously [6].

### 2.4. Evaluation of AMD Phenotype

AMD features were graded by clinicians and experienced graders on color fundus photographs using a modified version of the Wisconsin age-related maculopathy grading system [25]. Features were confirmed on optical coherence tomography (OCT) imaging and fundus autofluorescence (FAF) when available. GA was considered present when a sharply delineated round or oval area of RPE atrophy (≥0.024 mm^2^) with apparent choroidal vessels was visible, and when RPE atrophy together with a region of hypertransmission of at least 250 um in diameter and photoreceptor degeneration was visible on OCT [26]. CNV was considered present when a subretinal or sub-RPE neovascular membrane was visible, retinal scarring, subretinal hemorrhage, or serous RPE detachment were visible in combination with drusen and/or hard exudates on color fundus photographs and OCT. Early-intermediate AMD was characterized by the presence of soft drusen >125 um or reticular pseudodrusen, with or without retinal pigment changes, late AMD by GA, or CNV [27]. The clinical status was determined by the more severe eye.

### 2.5. Statistical Analysis

All statistical analyses were carried out using R, version 3.5.3 (R Core Team, 2016) and SPSS Statistics, version 24.0.0.1 (IBM, Armonk, NY, USA). Metabolite data were log(+1)-transformed to account for the very low levels of metabolites. Following the transformation, the levels were standardized to have a mean of zero and a standard deviation of one. To determine the associations of metabolite levels with early-intermediate AMD and late AMD, we age-matched the cases with controls, using MatchIt library in R. Age matching was carried out with exact match and gender matching where possible. Two controls for one AMD patient were matched where possible.

Metabolites were tested with univariate logistic regression analyses for early-intermediate AMD and late AMD as compared to no AMD, and late AMD as compared to early-intermediate AMD. Then, two approaches were taken to determine the associated metabolites. First, logistic regression analyses were performed on the pooled data set of matched cases and controls from all three study sites. Second, logistic regression analyses were performed per study site, and the results were combined into a meta-analysis. Obtained *p*-values from each step were corrected with FDR correction to account both for the correlation between metabolites and for multiple testing. FDR-corrected *p*-values that are lower than 0.05 were determined to be statistically significant associations. Only associations that were statistically significant in both the pooled analysis as well as the meta-analysis were eligible for subsequent analyses. Furthermore, effect estimate directions were checked for these shared results, and metabolites that did not show the same direction of effect for all three cohorts were removed from the results. We present the statistics of the meta-analysis results in this main text; however, all results of the individual cohort are provided in the Supplementary Tables S1–S3.

For incidence analyses, data from the Rotterdam Study were used. Participants aged 55 and older were followed up for AMD progression, underwent fundus examinations at baseline, and were re-examined every two to four years. For progression analyses, both data from EUGENDA and the Rotterdam Study were used. For incident and progression cases, follow-up ended at the first date of diagnosis or progression. Incidence or progression was assumed to have occurred halfway during the interval between two subsequent visits. Associations between metabolites and incidence of late AMD or progression from early and intermediate to late AMD were determined using a Cox proportional hazards regression adjusted for age, sex, and study site. Significant associations were visualized as the cumulative lifetime risk in Kaplan–Meier curves. Log-rank (Mantel–Cox) tests were performed to analyze the differences between groups.

Associations of the GRS, the pathway-specific GRS, smoking, and dietary intakes with metabolite levels were determined using a multivariable linear regression adjusted for age, sex, and study site. With respect to lifestyle factors, we additionally adjusted for body mass index (BMI). To study the independent effects of the GRS, LRS, and MRS on late stage AMD, a multivariable regression analysis was performed using a generalized linear model corrected for age and sex, with study site as a random factor. Using similar generalized linear models and the R mediation package, a causal mediation analysis was performed for the association between the GRS, LRS, and late AMD, with the MRS as the mediator.

Pathway analyses for AMD-associated metabolites were performed using MetaboAnalyst 5.0 (http://www.metaboanalyst.ca (accessed on 4 November 2022)), and included (1) an overrepresentation analysis and (2) a topology analysis. Overrepresentation analyses were based on the Kyoto Encyclopedia of Genes and Genomes-defined metabolic pathways (KEGG) database and Human Metabolome database (HMDB) as reference databases. Topology was based on the positional importance of the metabolites within the identified pathways. The analyses generated pathway impact scores and an associated *p*-value.

## 3. Results

A total of 3850 individuals were diagnosed with no AMD, 1114 individuals with early-intermediate AMD, and 959 individuals with late AMD (Table 1). The mean age of participants was 73.7 (SD 7.9) years; 41.4% were male. Early-intermediate AMD patients had a lower BMI (25.5 versus 26.0 kg/m2, *p*-value < 0.0001) and smoked less (11.9 versus 14.9%, *p*-value = 0.04) than the controls. Late AMD patients smoked more (15.2 versus 11.9%, *p*-value = 0.017), but had less hypertension (43.7 versus 34.3%, *p*-value < 0.0001) than the controls. AMD patients did not differ from others by diabetes type II status, after adjustment for age and sex. The characteristics for individuals per study site and for incidence and progression analyses are shown in Tables S4 and S5.

### 3.1. Metabolomic Profiles for AMD Disease Stages

**Figure 1:**
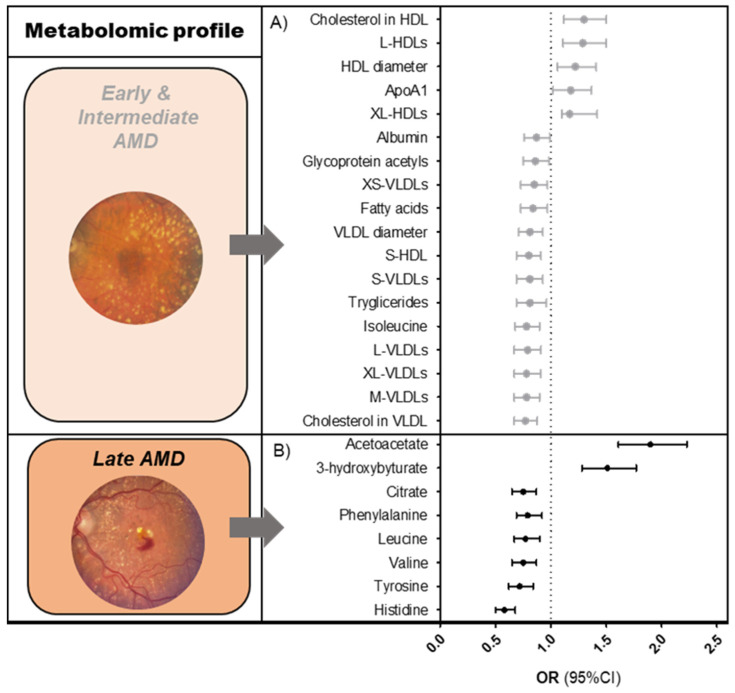
Metabolomic profiles of AMD. Graphs showing associations of Nightingale platform nuclear magnetic resonance (NMR) metabolites (**A**) with early and intermediate AMD, (**B**) with late AMD. Associations were determined using a logistic regression model, after matching cases and controls for age and sex. Reference = no AMD. The results shown are fixed estimates from our meta-analysis ordered according to effect size. Only metabolites that were FDR-significant in both the pooled analysis and meta-analysis were included. Several of the early-intermediate AMD-associated metabolites could be categorized within a single metabolite group. To simplify this, only one effect estimate (mean of effect estimates) is shown per metabolite group. More detailed results are shown in Tables S1 and S2. Abbreviations: AMD = age-related macular degeneration; OR = odds ratio; CI = confidence interval.

We identified 61 metabolites that were associated with early-intermediate AMD (Figure 1A, Table S1); of those, 94% of significant perturbations were lipid-related. Early-intermediate AMD patients had lower levels of all sizes of very low-density lipoprotein (VLDL) subparticles (ORs 0.8–0.9, *p*-valueFDR < 7.5 × 10^−3^), total- and monounsaturated fatty acids (ORs 0.9, *p*-valueFDR < 1.4 × 10^−2^), total serum triglycerides (OR 0.9, *p*-valueFDR = 1.6 × 10^−4^), and total cholesterol in VLDL (OR 0.9, *p*-valueFDR = 1.1 × 10^−4^). They had higher levels of total cholesterol in HDL/HDL2 (ORs 1.2, *p*-valueFDR < 7.6 × 10^−5^), large to extra-large HDL subparticles (ORs 1.1–1.2, *p*-valueFDR < 1.3 × 10^−4^), and apolipoprotein A1 (OR 1.2, *p*-valueFDR < 1.3 × 10^−3^). Non-lipid-related perturbations in early-intermediate AMD patients included lower levels of citrate, albumin, glycoprotein acetyls, and the amino acid isoleucine (ORs 0.9, *p*-valueFDR < 1.4 × 10^−2^).

We identified eight metabolites that were significantly associated with late AMD (Figure 1B, Table S2). Late AMD patients had lower levels of the amino acids histidine, tyrosine, valine, leucine, and phenylalanine (ORs 0.6–0.8, *p*-valueFDR < 1.5 × 10^−3^), lower levels of citrate (OR 0.8, *p*-valueFDR = 2.7 × 10^−3^), and higher levels of the ketone bodies acetoacetate and 3-hydroxybutyrate (OR 1.4, *p*-valueFDR < 2.5 × 10^−8^) as compared to the healthy controls.

When comparing metabolite levels between the two AMD stages, citrate was the only metabolite that was associated with both, although it was less significant in the early-intermediate AMD analysis (OR 0.9, *p*-valueFDR = 1.4 × 10^−2^) (Table S3). We found that 6/8 metabolites for late AMD (i.e., amino acids histidine, tyrosine, and leucine, and ketone bodies 3-hydroxybutyrate and acetoacetate) remained significant in the late AMD versus early-intermediate AMD analysis, with similar effect estimates as compared with the late AMD versus no AMD analysis (*p*-valueFDR < 1.7 × 10^−2^). Additional metabolites in the late AMD vs. early-intermediate AMD analysis were also found. Compared to early-intermediate AMD, patients with late AMD had lower levels of the amino acid glutamine, lower medium to large HDLs, and lower apolipoprotein A1, while they had higher levels of both small VLDLs and glycoprotein acetyls (*p*-valueFDR < 4.7 × 10^−2^).

Incidence analyses were performed using the Rotterdam Study cohorts. During an observation time of 15 years, 374 developed incident early-intermediate AMD within on average 6.0 years (SD 5.2); 88 persons developed incident late AMD within on average 11.2 years (SD 1.2). Although no metabolites reached the FDR significance level, effect- estimates and directions were similar to those found in cross-sectional analyses (Tables S6 and S7). Using pooled data from the Rotterdam and EUGENDA cohorts, we compared metabolite levels between patients who had early-intermediate AMD but who progressed to late AMD (*n* = 89) during a mean follow-up of 6.0 years (SD 5.2 years) with those who remained stable (*n* = 182). Both the fluid balance-related albumin (HR 0.57, 95%CI 0.44–0.73, *p*-valueFDR = 1.0 × 10^−5^) and the glycolysis-related citrate (HR 0.61, 95%CI 0.44–0.84, *p*-valueFDR = 5.0 × 10^−3^) were inversely associated with a risk of progression. Likewise, lower levels of both metabolites showed a higher cumulative risk of progression (Figure S2).

To explore the biologic mechanisms related to metabolites, we performed a pathway analysis using MetaboAnalyst 5.0. We identified three different pathways that passed the FDR correction for metabolites associated with late AMD, i.e., biosynthesis of phenylalanine, tyrosine, and tryptophan (*p*-valueFDR = 5.8 × 10^−3^); synthesis and degradation of ketone bodies (*p*-valueFDR = 6.5 × 10^−3^); and metabolism of phenylalanine (*p*-valueFDR = 1.4 × 10^−2^) (Table S8). This metabolic pathway analysis did not reveal a more overarching biological process.

As low systemic amino acid and high ketone body levels may indicate a low glucose availability in the bloodstream, we also investigated fasting serum glucose levels and diabetes type 2 status in our RS cohorts. Mean fasting serum glucose levels and diabetes type 2 status did not differ between early-intermediate AMD patients or late AMD patients and controls (*p*-value = 0.830, *p*-value = 0.809). In addition, fasting serum glucose levels did not show any linear relationship with amino acid or ketone body levels (Table S9, Figure S3).

### 3.2. Association of Genetic and Lifestyle Factors with Metabolites

We subsequently determined associations of the GRS and lifestyle factors with metabolite measurements. Associations were adjusted for age, sex, and study site; associations with lifestyle factors were additionally adjusted for BMI. Detailed results are shown separately for early-intermediate and late AMD metabolites (Figures S4 and S5).

The total GRS was associated with 47 out of the 69 identified metabolites; in particular with higher levels of medium to extra-large HDL subclasses, cholesterols in HDL, and fatty acids (betas ≅ 0.02–0.03, *p*-valueFDR < 3.4 × 10^−5^), and with lower levels of VLDL subclasses, small HDLs, and fatty acids (betas ≅ −0.03 to 0.04, *p*-valueFDR < 2.2 × 10^−6^) (Tables S10 and S11). Pathway analyses showed that the complement-based GRS was associated with lower levels of histidine (beta −0.04, *p*-valueFDR = 1.6 × 10^−2^) and higher levels of phenylalanine, 3-hydroxybutyrate, and citrate (betas ≅ 0.03, *p*-valueFDR < 2.2 × 10^−2^). The lipid-based GRS was associated with 16 lipid-related metabolites, i.e., with lower levels of extra-small to medium VLDLs, small to medium HDLs, total fatty acids, cholesterol in VLDL, and triglycerides in HDL (betas ≅ −0.04 to −0.11, *p*-valueFDR < 1.8 × 10^−2^), and with higher levels of medium HDLs and a mean diameter VLDL (betas ≅ 0.03 to 0.04, *p*-valueFDR < 3.7 × 10^−2^). A high-risk ARMS2/HTRA1 genotype was associated with higher levels of the ketone body acetoacetate, phenylalanine, and citrate (betas ≅ 0.03–0.05, *p*-valueFDR < 5.0 × 10^−3^). The ECM-based GRS was not associated with any change in metabolite levels. Estimates and directions did not change after the GRS pathways were corrected for other variants.

The LRS representing total lifestyle score was associated with 30 out of the 69 identified metabolites; most significantly with higher levels of monounsaturated fatty acids and the inflammation-related glycoprotein acetyls (betas ≅ 0.07–0.08, *p*-valueFDR < 1.4 × 10^−4^) (Tables S10 and S11). Of the individual lifestyle factors, smoking was associated with eight metabolites; particularly with lower levels of citrate (beta −0.09, *p*-valueFDR = 3.1 × 10^−6^) and albumin (beta −0.10, *p*-valueFDR = 2.9 × 10^−5^), but also with lower levels of the amino acids valine and histidine (betas ≅ −0.05 to −0, *p*-valueFDR < 2.7 × 10^−2^). Smoking was also associated with higher levels of monounsaturated fatty acids, glycoprotein acetyls, and small HDLs (betas ≅ 0.08 to 0.10, *p*-valueFDR < 9.0 × 10^−3^). With respect to favorable lifestyle factors, vegetable intake was associated with 49 metabolites, i.e., with lower levels of the VLDL subclasses, fatty acids, cholesterols, apolipoproteins, and the ketone body 3-hydroxybutyrate (−0.18 to −0.30, *p*-valueFDR < 4.0 × 10^−2^), and higher levels of large HDLs (betas ≅ 0.16–0.20, *p*-valueFDR < 0.05). Fruit intake was particularly associated with lower levels of 3-hydroxybutyrate (beta −0.26, *p*-valueFDR = 4.9 × 10^−6^), and also with lower levels of acetoacetate, phenylalanine, tyrosine, and of citrate (betas ≅ −0.13 to −0.16, *p*-valueFDR < 1.8 × 10^−2^). Finally, fish intake was associated with higher levels of the amino acids valine and tyrosine (betas ≅ 0.25–0.29, *p*-valueFDR < 8.0 × 10^−3^).

### 3.3. Interplay between Metabolite, Genetic, and Lifestyle Factors for Late AMD

To investigate the independent contribution of these targeted blood NMR metabolites to late AMD, we calculated a metabolomic risk score (MRS) using the eight metabolites (citrate, histidine, phenylalanine, tyrosine, valine, 3-hydroxybutyrate, and acetoacetate) identified for late AMD. We then performed a multivariable analysis of late AMD where the GRS, LRS, and MRS were included as determinants. The MRS remained associated with a higher odds of late AMD (OR 1.6, 95%CI 1.4–1.9, *p*-value = 3.5 × 10^−9^) after correction for the GRS and LRS, as well as age, sex, and study site (Table S12). Using the risk scores, we also analyzed the mediating effect of the MRS on the association of LRS and GRS with late AMD. Of the effect of the LRS on late AMD, 19.5% (95%CI 7.0–88.0) was mediated by the MRS after correction for the GRS (Figure S6). For the GRS, only 5.3% (95%CI 2.6–9.0) of the effect on late AMD was mediated by the MRS.

## 4. Discussion

**Figure 2:**
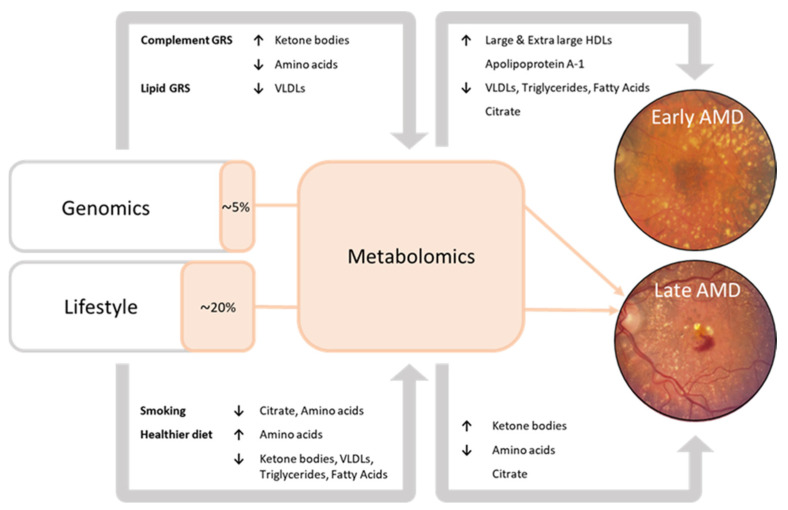
Summary of the results. Associations of blood metabolites with early-intermediate AMD and late AMD. Associations of the genetic risk score pathways with blood metabolites. Associations of the lifestyle factors with blood metabolites. Orange sections represent the mediating effect of the metabolomic risk score on the association of the genetic risk score and lifestyle risk score with late AMD (% = the proportion of association of genetics and lifestyle with late AMD that can be attributed to the Nightingale blood metabolites).

In this study, we performed the largest investigation into systemic metabolomic profiles associated with age-related macular degeneration (AMD) to date. To our knowledge, this report is the first to demonstrate the interplay between genetics, lifestyle, and blood metabolites in the development towards late AMD. A summary of the results is shown in Figure 2.

Late AMD patients had higher levels of ketone bodies (acetoacetate, 3-hydroxybutyrate), but lower levels of amino acids (histidine, leucine, valine, tyrosine, phenylalanine) as compared to the healthy controls. In early to intermediate AMD patients, 94% of perturbations were lipid-related, with higher levels of large to extra-large HDL subparticles and apolipoprotein A1, and lower levels of VLDLs, triglycerides, and fatty acids. A favorable lifestyle characterized by a healthy diet was associated with higher levels of amino acids and lower levels of ketone body levels, an unfavorable lifestyle, including current smoking, showed opposite effects. The data of this study also suggest that metabolite levels implicated in late AMD predominantly reflect lifestyle; blood metabolites mediated 20% of the effect of lifestyle factors on late AMD, and only 5% of the effect of the genetic factors.

So far, studies have identified over 100 blood metabolites associated with AMD [11,28]. In a previous report by Acar et al., we identified 60 metabolites for any AMD, of which 89% were lipid-related [21]. The current report finds novel associations with late AMD: inverse associations with the amino acids histidine and valine, and positive associations with the ketone bodies acetoacetate and 3-hydroxybyturate. For early-intermediate AMD, we also found associations not described before: inverse associations with albumin, glycoprotein acetyls, and positive associations with apolipoprotein A1 and other HDLs. The current study adds to the understanding that blood metabolite profiles vary with AMD severity. Lains et al. also found linear trends across the stages for 67 plasma metabolites in 90 patients with early, intermediate, and late AMD. [29] While a different metabolomics platform was used, similar profiles were established with perturbations in mostly lipids and amino acids [12]. Urine samples of AMD patients validated the depletion of amino acids (valine, tyrosine) and citrate [30].

Insight into metabolic signatures may identify novel biological pathways. We found lower levels of amino acids and citrate, and higher levels of ketone bodies in the blood samples of late AMD patients. Several mechanisms may explain these findings. Amino acids are organic substances that play a major role in several biological processes including protein synthesis, regulation of hormone secretion, gene expression, and cell signaling. Of the amino acids associated with late AMD, histidine, leucine, phenylalanine, and valine are considered “essential”. They cannot be synthesized de novo by the body and must be provided from the diet to meet optimal requirements. Only tyrosine is considered “non-essential” and can be synthesized by the body in adequate amounts. Functionally, histidine is involved in the production of erythrocytes and leukocytes and the maintenance of acidic balance in the body. Leucine is involved in muscle growth and repair [30]. Phenylalanine and tyrosine are precursors for several neurotransmitters including dopamine, norepinephrine, and epinephrine. Overall, low levels of essential amino acids in the systemic circulation are associated with enhanced energy requirement, subclinical nutritional deficiency, and lower body muscle mass [30,31]. Changes in amino acid concentrations may also have local effects in the eye. Leucine and valine are known to activate and synthesize glutamate and glutamine; compounds that are critical for neurotransmission and metabolism in the retina [32,33,34]. Ketone bodies, moreover, are water-soluble breakdown products from fatty acids produced by the liver during times of low food intake, carbohydrate restriction, excessive alcohol consumption, and uncontrolled diabetes mellitus type I; they have multi-dimensional roles such as energy fuels for extra-hepatic tissues, signal mediation, and modulation of inflammation and oxidative stress. Acetoacetate and 3-hydroxybutyrate are the most important ketone bodies as they are the primary source of energy for the body during periods of ketosis. 3-hydroxybutyrate is the main ketone body used by the brain and other organs for fuel, while acetoacetate is primarily used by the muscles for energy. In the retina, 3-hydroxybutyrate appears to prevent accumulation of lipids due to oxidative stress [35,36] [21,37,38,39] [40]. How systemic levels of ketone bodies that are high but still within physiologic range associate with especially late stages of AMD is intriguing but not easily explained. While low amino acid levels and high ketone body levels may point toward decreased availability of glucose in the blood stream, we did not find any differences in fasting glucose levels or diabetic status across AMD stages. 

The perturbations of lipoprotein, apolipoprotein, fatty acid, and cholesterol levels found in early to intermediate AMD patients are not unexpected. Numerous biochemical and histological studies revealed that drusen have significant lipid composition, and epidemiological studies have already suggested that there may be an important link between systemic dyslipidemia and drusen formation, especially with HDL [41]. In support, drusenomic studies recently showed that a considerable proportion of drusen proteins (>60%) uniquely originate from the blood, proposing a “meet, greet, and stick” hypothesis, where drusen components accumulate to form large deposits between the RPE and Bruch’s membrane [19]. Particularly, apolipoproteins, including ApoA1 and the protein-groups related to the LDL, HDL, and VLDL metabolism, but also albumin, were deemed likely to be derived from the blood, and not from the retina.

We found low albumin levels particularly in patients with progressive AMD. A well-known association with low levels of albumin in the systemic circulation is malnutrition, which may correspond to an unfavorable diet that appears to be more common in these patients. Other potential explanations for low levels of this fluid-balance protein are an increase in oxidative stress and inflammatory responses [42,43].

An important question in the context of this study is how plasma proteins and metabolites find their way into the space between the RPE and the basal lamina of the RPE. It is plausible that some proteins and metabolites exit the choroidal vessels into the extracellular space adjacent to the RPE, especially as the basal lamina of the RPE becomes compromised with age [19]. Still, functional studies that demonstrate the effects of systemic changes on retinal immunity, inflammation, and architecture are of vital importance.

The integration of metabolomic, genetic, and lifestyle factors in a systems biology approach holds great potential for elucidating biological processes involved in AMD. Nevertheless, such multilevel integration has been a vital challenge in the AMD research field [44]. In this study, we identified several pathways that affect the systemic regulation of AMD-relevant metabolites (S10-S11). Risk variants in complement genes and in ARMS2/HTRA1 [3,6] were associated with lower levels of the amino acids histidine and phenylalanine and higher levels of the ketone bodies acetoacetate and 3-hydroxybutyrate. In addition, variants in CETP, LIPC, ABCA1, and APOE, were associated mostly with lipid-related metabolites, particularly with lower levels of VLDL and higher levels of medium to extra-large HDL levels [6,18,21,28]. We also observed that a large proportion of AMD relevant metabolites was associated with lifestyle factors [8,9,10,45]; altogether, current smoking and intake of vegetables, fruits, and fish was associated with over 70% of all AMD-relevant metabolites. Our data additionally suggest that metabolites can be used to monitor lifestyle changes in AMD patients; approximately 20% of the effect of lifestyle changes on late AMD were explained by perturbations in the blood. However, a large proportion of lifestyle risk remains unexplained. Untargeted investigations using robust mass spectrometry or NMR will likely result in more comprehensive metabolic associations. Finally, our data shows that the association between the MRS and late AMD was still present after correction for the GRS and LRS. While it is promising that metabolites may have independent effects on AMD, we could not assess to what extent rare genetic variants or other AMD-related dietary or lifestyle factors, might have influenced our results. We can also not exclude the possibility of residual confounding.

The results need to be interpreted in light of several strengths and limitations. A strength is the availability of metabolite measurements in 5923 samples, which makes it the largest metabolomic profiling study performed for AMD so far. Criteria for genetic, lifestyle, metabolomics, and phenotype data were carefully harmonized across studies. The large sample size of our study allowed for the analysis of various common genetic, lifestyle, and metabolite factors in association with AMD. In addition, the availability of prospective data in the Rotterdam Study cohorts enabled us to investigate the effects of metabolite levels on AMD progression over time. Our study also had limitations. Only a small number of incident end-points of AMD were available for analysis. However, the effect estimates showed a similar size and effect when compared to the cross-sectional analyses. As briefly mentioned, the NMR-based targeted metabolomics platform involves only a limited number of measurements compared to mass spectrometry-based approaches. Analysis of other metabolic biomarker platforms will likely result in the identification of additional biomarkers, improved early-late AMD discrimination, and improved analysis of genetic and lifestyle effects on metabolite levels. Nevertheless, the NMR platform is widely considered an affordable, well-standardized, and high-throughput platform. In this study, the platform allowed for direct quantifications of metabolites that were consistent between batches and cohorts.

## 5. Conclusions

In conclusion, we identified distinct blood metabolomic profiles for various stages of AMD. Amino acids and ketone bodies were associated with late AMD and lipid-related metabolites with early and intermediate stages of AMD. This study highlights the impact of genetic and lifestyle factors on circulating metabolites, and suggests that they could potentially be used to monitor AMD progression in patients. Future studies should clarify whether the metabolites play a causal role in AMD pathogenesis or are merely temporary passersby.

## Figures and Tables

**Table 1:** Baseline characteristics of the included participants.

	Subjects in the Analysis * (*n* = 5923)				
	Controls (*n* = 3850)	Early-Intermediate AMD (*n* = 1114)	Late AMD (*n* = 959)	*p*-Value Early-Intermediate AMD	*p*-Value Late AMD
Baseline age (mean ± SD)	72.4 ± 7.2	74.7 ± 8.1	77.7 ± 8.2	<0.0001	<0.0001
Sex (% male)	41.8	40	41.5	0.287	0.842
BMI	*n* = 3526	*n* = 970	*n* = 805		
mean ± SD	26.0 ± 3.6	25.5 ± 3.5	26.0 ± 3.8	<0.0001	0.772
Smoking	*n* = 3583	*n* = 990	*n* = 808		
Former	47.5	47.1	49.6	0.317	0.07
Current	14.9	11.9	15.2	0.04	0.017
Hypertension	*n* = 3610	*n* = 1011	*n* = 878		
% yes	43.7	41.9	34.3	0.055	<0.0001
Diabetes type II	*n* = 3450	*n* = 992	*n* = 869		
% yes	9.8	9.3	13	0.346	0.088

* Subjects who had data on serum measurements and AMD phenotype. *p*-values based on regression analyses adjusted for age and sex. Abbreviations: AMD = age-related macular degeneration; BMI = body mass index; SD = standard deviation.

## Data Availability

Data sharing not applicable. No new data were created or analyzed in this study.

## Supplementary Materials

The following supporting information can be downloaded at: https://www.mdpi.com/article/10.3390/metabo13060701/s1, Figure S1: Flowchart of the inclusion and exclusion of subjects; Figure S2: Cumulative risk of progression from early-intermediate AMD to late AMD; Figure S3: Scatter plot to assess the linearity between fasting serum glucose levels and late-AMD associated metabolite levels; Figure S4: Associations between genetic factors, lifestyle factors, and metabolite levels for late stage AMD; Figure S5: Associations between genetic factors, lifestyle factors, and metabolite levels (per metabolite group) for early- intermediate stage AMD; Figure S6: Metabolomic risk score (MRS) as a mediator in the association between lifestyle risk and late AMD; Table S1: Associations between metabolites and early and intermediate AMD; Table S2. Associations between metabolites and late AMD; Table S3. Associations between metabolites and late AMD; Table S4. Baseline characteristics per study site; Table S5. Baseline characteristics for subjects in incidence and progression analyses; Table S6. Associations between metabolites and late AMD; Table S7. Associations between metabolites and early-intermediate AMD; Table S8. Pathway analysis of significant metabolites in late AMD; Table S9. Association of fasting serum glucose and diabetes with AMD stage; Tables S10 and S11. Associations between the GRS, LRS, and metabolites; Table S12. Association of genetic, lifestyle, and metabolite risk scores with late AMD.

## References

[1] Blindness G.B.D., Vision Impairment C., Vision Loss Expert Group of the Global Burden of Disease Study (2021). Causes of blindness and vision impairment in 2020 and trends over 30 years, and prevalence of avoidable blindness in relation to VISION 2020: The Right to Sight: An analysis for the Global Burden of Disease Study. Lancet Glob. Health.

[2] Colijn J.M., Buitendijk G.H.S., Prokofyeva E., Alves D., Cachulo M.L., Khawaja A.P., Cougnard-Gregoire A., Merle B.M.J., Korb C., Erke M.G. (2017). Prevalence of Age-Related Macular Degeneration in Europe: The Past and the Future. Ophthalmology.

[3] Fritsche L.G., Igl W., Bailey J.N., Grassmann F., Sengupta S., Bragg-Gresham J.L., Burdon K.P., Hebbring S.J., Wen C., Gorski M. (2016). A large genome-wide association study of age-related macular degeneration highlights contributions of rare and common variants. Nat. Genet..

[4] Geerlings M.J., de Jong E.K., den Hollander A.I. (2017). The complement system in age-related macular degeneration: A review of rare genetic variants and implications for personalized treatment. Mol. Immunol..

[5] Han X., Gharahkhani P., Mitchell P., Liew G., Hewitt A.W., MacGregor S. (2020). Genome-wide meta-analysis identifies novel loci associated with age-related macular degeneration. J. Hum. Genet..

[6] Colijn J.M., Meester-Smoor M., Verzijden T., de Breuk A., Silva R., Merle B.M.J., Cougnard-Gregoire A., Hoyng C.B., Fauser S., Coolen A. (2021). Genetic Risk, Lifestyle, and Age-Related Macular Degeneration in Europe: The EYE-RISK Consortium. Ophthalmology.

[7] de Breuk A., Acar I.E., Kersten E., Schijvenaars M., Colijn J.M., Haer-Wigman L., Bakker B., de Jong S., Meester-Smoor M.A., Verzijden T. (2020). Development of a Genotype Assay for Age-Related Macular Degeneration: The EYE-RISK Consortium. Ophthalmology.

[8] Merle B.M., Silver R.E., Rosner B., Seddon J.M. (2015). Adherence to a Mediterranean diet, genetic susceptibility, and progression to advanced macular degeneration: A prospective cohort study. Am. J. Clin. Nutr..

[9] Merle B.M.J., Colijn J.M., Cougnard-Gregoire A., de Koning-Backus A.P.M., Delyfer M.N., Kiefte-de Jong J.C., Meester-Smoor M., Feart C., Verzijden T., Samieri C. (2019). Mediterranean Diet and Incidence of Advanced Age-Related Macular Degeneration: The EYE-RISK Consortium. Ophthalmology.

[10] de Koning-Backus A.P.M., Buitendijk G.H.S., Kiefte-de Jong J.C., Colijn J.M., Hofman A., Vingerling J.R., Haverkort E.B., Franco O.H., Klaver C.C.W. (2019). Intake of Vegetables, Fruit, and Fish is Beneficial for Age-Related Macular Degeneration. Am. J. Ophthalmol..

[11] Hou X.W., Wang Y., Pan C.W. (2020). Metabolomics in Age-Related Macular Degeneration: A Systematic Review. Investig. Ophthalmol. Vis. Sci..

[12] Lains I., Kelly R.S., Miller J.B., Silva R., Vavvas D.G., Kim I.K., Murta J.N., Lasky-Su J., Miller J.W., Husain D. (2018). Human Plasma Metabolomics Study across All Stages of Age-Related Macular Degeneration Identifies Potential Lipid Biomarkers. Ophthalmology.

[13] Lains I., Gantner M., Murinello S., Lasky-Su J.A., Miller J.W., Friedlander M., Husain D. (2019). Metabolomics in the study of retinal health and disease. Prog. Retin. Eye Res..

[14] Brown C.N., Green B.D., Thompson R.B., den Hollander A.I., Lengyel I., Eye-Risk Consortium (2018). Metabolomics and Age-Related Macular Degeneration. Metabolites.

[15] Osborn M.P., Park Y., Parks M.B., Burgess L.G., Uppal K., Lee K., Jones D.P., Brantley M.A. (2013). Metabolome-wide association study of neovascular age-related macular degeneration. PLoS ONE.

[16] Kersten E., Dammeier S., Ajana S., Groenewoud J.M.M., Codrea M., Klose F., Lechanteur Y.T., Fauser S., Ueffing M., Delcourt C. (2019). Metabolomics in serum of patients with non-advanced age-related macular degeneration reveals aberrations in the glutamine pathway. PLoS ONE.

[17] Lambert V., Hansen S., Schoumacher M., Lecomte J., Leenders J., Hubert P., Herfs M., Blacher S., Carnet O., Yip C. (2020). Pyruvate dehydrogenase kinase/lactate axis: A therapeutic target for neovascular age-related macular degeneration identified by metabolomics. J. Mol. Med..

[18] Lains I., Zhu S., Han X., Chung W., Yuan Q., Kelly R.S., Gil J.Q., Katz R., Nigalye A., Kim I.K. (2021). Genomic-Metabolomic Associations Support the Role of LIPC and Glycerophospholipids in Age-Related Macular Degeneration. Ophthalmol. Sci..

[19] Bergen A.A., Arya S., Koster C., Pilgrim M.G., Wiatrek-Moumoulidis D., van der Spek P.J., Hauck S.M., Boon C.J.F., Emri E., Stewart A.J. (2019). On the origin of proteins in human drusen: The meet, greet and stick hypothesis. Prog. Retin. Eye Res..

[20] Kersten E., Paun C.C., Schellevis R.L., Hoyng C.B., Delcourt C., Lengyel I., Peto T., Ueffing M., Klaver C.C.W., Dammeier S. (2018). Systemic and ocular fluid compounds as potential biomarkers in age-related macular degeneration. Surv. Ophthalmol..

[21] Acar I.E., Lores-Motta L., Colijn J.M., Meester-Smoor M.A., Verzijden T., Cougnard-Gregoire A., Ajana S., Merle B.M.J., de Breuk A., Heesterbeek T.J. (2020). Integrating Metabolomics, Genomics, and Disease Pathways in Age-Related Macular Degeneration: The EYE-RISK Consortium. Ophthalmology.

[22] Delcourt C., Korobelnik J.F., Buitendijk G.H., Foster P.J., Hammond C.J., Piermarocchi S., Peto T., Jansonius N., Mirshahi A., Hogg R.E. (2016). Ophthalmic epidemiology in Europe: The “European Eye Epidemiology” (E3) consortium. Eur. J. Epidemiol..

[23] Heesterbeek T.J., de Jong E.K., Acar I.E., Groenewoud J.M.M., Liefers B., Sanchez C.I., Peto T., Hoyng C.B., Pauleikhoff D., Hense H.W. (2019). Genetic risk score has added value over initial clinical grading stage in predicting disease progression in age-related macular degeneration. Sci. Rep..

[24] Biarnes M., Colijn J.M., Sousa J., Ferraro L.L., Garcia M., Verzijden T., Meester-Smoor M.A., Delcourt C., Klaver C.C.W., den Hollander A.I. (2020). Genotype- and Phenotype-Based Subgroups in Geographic Atrophy Secondary to Age-Related Macular Degeneration: The EYE-RISK Consortium. Ophthalmol. Retin..

[25] Gattoussi S., Buitendijk G.H.S., Peto T., Leung I., Schmitz-Valckenberg S., Oishi A., Wolf S., Deak G., Delcourt C., Klaver C.C.W. (2019). The European Eye Epidemiology spectral-domain optical coherence tomography classification of macular diseases for epidemiological studies. Acta Ophthalmol..

[26] Guymer R.H., Rosenfeld P.J., Curcio C.A., Holz F.G., Staurenghi G., Freund K.B., Schmitz-Valckenberg S., Sparrow J., Spaide R.F., Tufail A. (2020). Incomplete Retinal Pigment Epithelial and Outer Retinal Atrophy in Age-Related Macular Degeneration: Classification of Atrophy Meeting Report 4. Ophthalmology.

[27] Thee E.F., Meester-Smoor M.A., Luttikhuizen D.T., Colijn J.M., Enthoven C.A., Haarman A.E.G., Rizopoulos D., Klaver C.C.W., EyeNED Reading Center (2020). Performance of Classification Systems for Age-Related Macular Degeneration in the Rotterdam Study. Transl. Vis. Sci. Technol..

[28] Han X., Ong J.S., Hewitt A.W., Gharahkhani P., MacGregor S. (2021). The effects of eight serum lipid biomarkers on age-related macular degeneration risk: A Mendelian randomization study. Int. J. Epidemiol..

[29] Lains I., Chung W., Kelly R.S., Gil J., Marques M., Barreto P., Murta J.N., Kim I.K., Vavvas D.G., Miller J.B. (2019). Human Plasma Metabolomics in Age-Related Macular Degeneration: Meta-Analysis of Two Cohorts. Metabolites.

[30] Lains I., Duarte D., Barros A.S., Martins A.S., Carneiro T.J., Gil J.Q., Miller J.B., Marques M., Mesquita T.S., Barreto P. (2019). Urine Nuclear Magnetic Resonance (NMR) Metabolomics in Age-Related Macular Degeneration. J. Proteome Res..

[31] Kettunen J., Tukiainen T., Sarin A.P., Ortega-Alonso A., Tikkanen E., Lyytikainen L.P., Kangas A.J., Soininen P., Wurtz P., Silander K. (2012). Genome-wide association study identifies multiple loci influencing human serum metabolite levels. Nat. Genet..

[32] Wu G. (2009). Amino acids: Metabolism, functions, and nutrition. Amino Acids.

[33] Durante W. (2020). Amino Acids in Circulatory Function and Health. Adv. Exp. Med. Biol..

[34] Kalloniatis M., Loh C.S., Acosta M.L., Tomisich G., Zhu Y., Nivison-Smith L., Fletcher E.L., Chua J., Sun D., Arunthavasothy N. (2013). Retinal amino acid neurochemistry in health and disease. Clin. Exp. Optom..

[35] Adijanto J., Du J., Moffat C., Seifert E.L., Hurle J.B., Philp N.J. (2014). The retinal pigment epithelium utilizes fatty acids for ketogenesis. J. Biol. Chem..

[36] Reyes-Reveles J., Dhingra A., Alexander D., Bragin A., Philp N.J., Boesze-Battaglia K. (2017). Phagocytosis-dependent ketogenesis in retinal pigment epithelium. J. Biol. Chem..

[37] Wurtz P., Havulinna A.S., Soininen P., Tynkkynen T., Prieto-Merino D., Tillin T., Ghorbani A., Artati A., Wang Q., Tiainen M. (2015). Metabolite profiling and cardiovascular event risk: A prospective study of 3 population-based cohorts. Circulation.

[38] Burgess S., Davey Smith G. (2017). Mendelian Randomization Implicates High-Density Lipoprotein Cholesterol-Associated Mechanisms in Etiology of Age-Related Macular Degeneration. Ophthalmology.

[39] Colijn J.M., den Hollander A.I., Demirkan A., Cougnard-Gregoire A., Verzijden T., Kersten E., Meester-Smoor M.A., Merle B.M.J., Papageorgiou G., Ahmad S. (2019). Increased High-Density Lipoprotein Levels Associated with Age-Related Macular Degeneration: Evidence from the EYE-RISK and European Eye Epidemiology Consortia. Ophthalmology.

[40] Umpierrez G.E., DiGirolamo M., Tuvlin J.A., Isaacs S.D., Bhoola S.M., Kokko J.P. (2000). Differences in metabolic and hormonal milieu in diabetic- and alcohol-induced ketoacidosis. J. Crit. Care.

[41] Lin J.B., Halawa O.A., Husain D., Miller J.W., Vavvas D.G. (2022). Dyslipidemia in age-related macular degeneration. Eye.

[42] Artigas A., Wernerman J., Arroyo V., Vincent J.L., Levy M. (2016). Role of albumin in diseases associated with severe systemic inflammation: Pathophysiologic and clinical evidence in sepsis and in decompensated cirrhosis. J. Crit. Care.

[43] Cho A.R., Lee S.B., Hong K.W., Jung D.H. (2021). C-reactive protein-to-albumin ratio and 8-year incidence of type 2 diabetes: The Korean genome and epidemiology study. Acta Diabetol..

[44] Shah S.H., Newgard C.B. (2015). Integrated metabolomics and genomics: Systems approaches to biomarkers and mechanisms of cardiovascular disease. Circ. Cardiovasc. Genet..

[45] Scalbert A., Brennan L., Manach C., Andres-Lacueva C., Dragsted L.O., Draper J., Rappaport S.M., van der Hooft J.J., Wishart D.S. (2014). The food metabolome: A window over dietary exposure. Am. J. Clin. Nutr..

