# Supplementary material for: Systemic Metabolomics in a Framework of Genetics and Lifestyle in Age-Related Macular Degeneration
Source: Metabolites. 2023 May 27;13(6):701. doi: 10.3390/metabo13060701 (PMC10305219; doi:10.3390/metabo13060701)
Supplement: Supplementary file 1 [file metabolites-13-00701-s001.zip › metabolites-2384753-supplementary-1.pdf]

Supplementary File

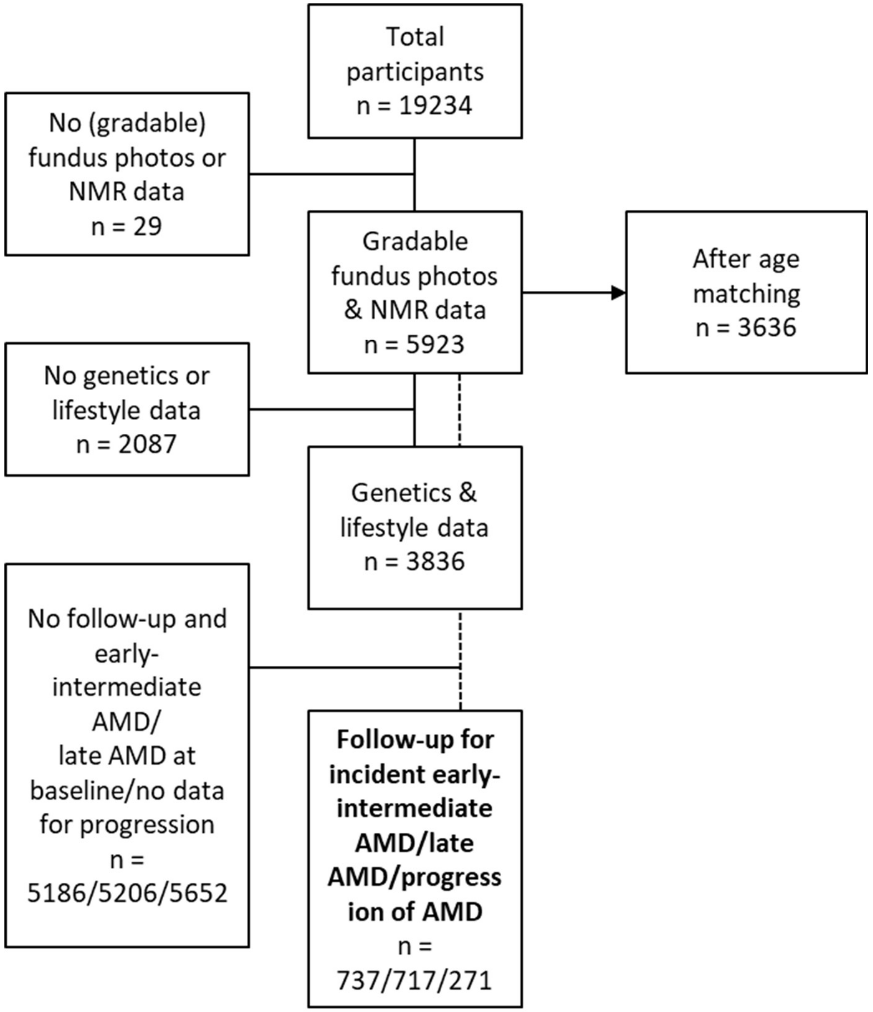

Figure S1. Flowchart of inclusion and exclusion of subjects

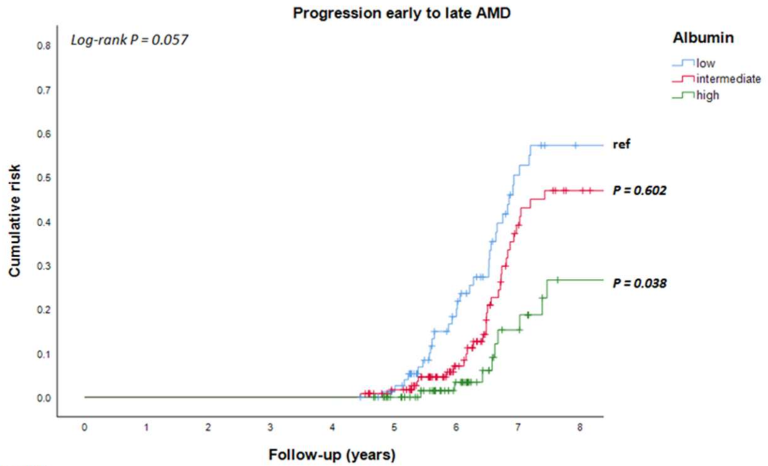

Number at risk

Low	80	80	80	80	80	74	46	22	16
Intermediate	123	123	123	123	123	112	69	31	23
high	84	84	84	84	84	76	50	26	17

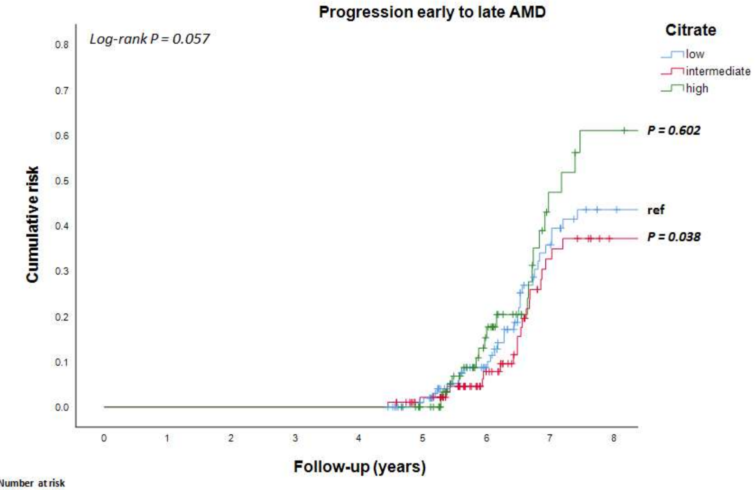

Number at risk

Low	110	110	110	110	110	100	68	35	25
Intermediate	99	99	99	99	99	89	57	30	23
high	72	72	72	72	72	67	35	12	8

Figure S2. Cumulative risk of progression from early-intermediate AMD to late AMD.

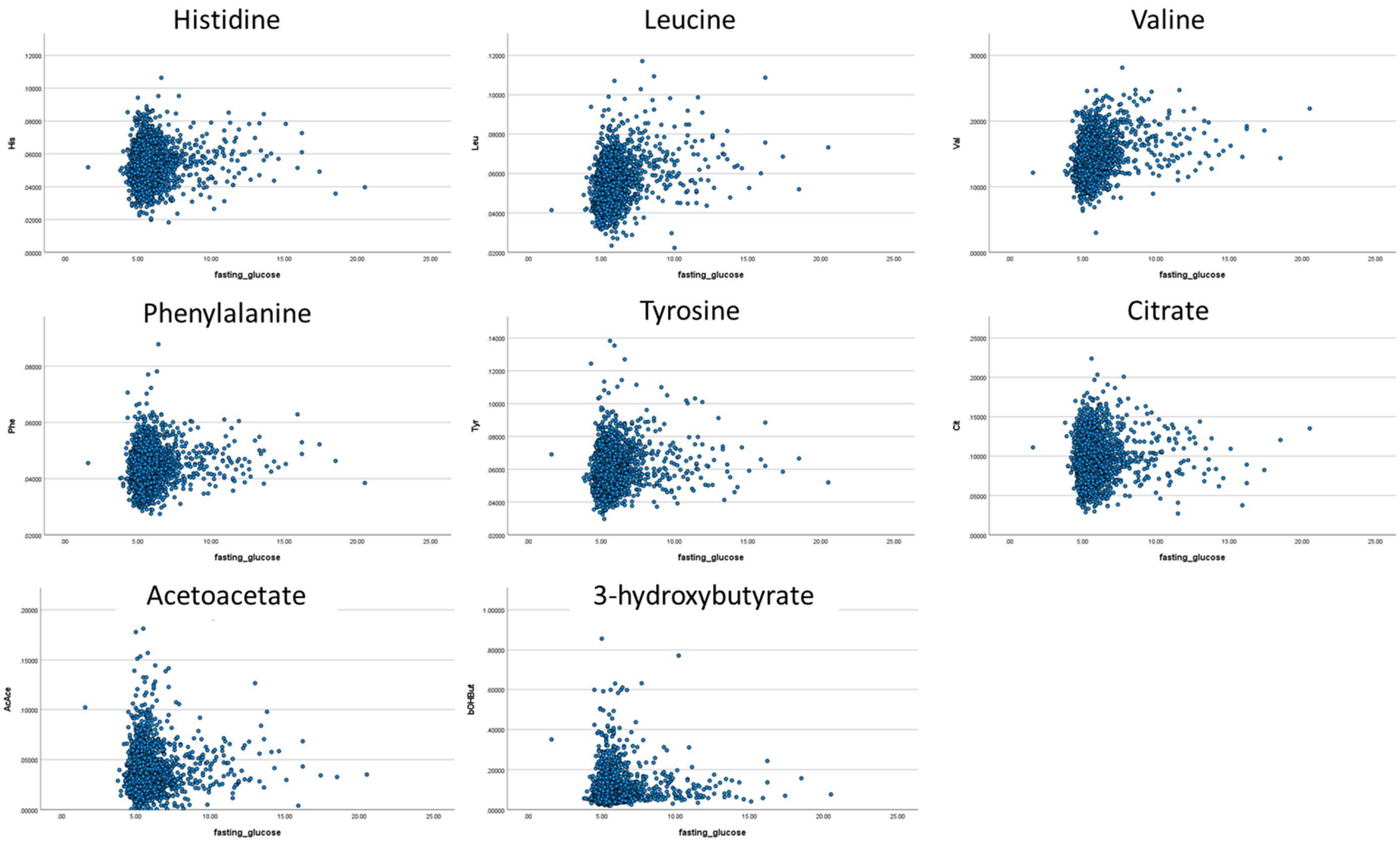

Figure S3. Scatter plot to assess linearity between fasting serum glucose levels and late-AMD associated metabolite levels.

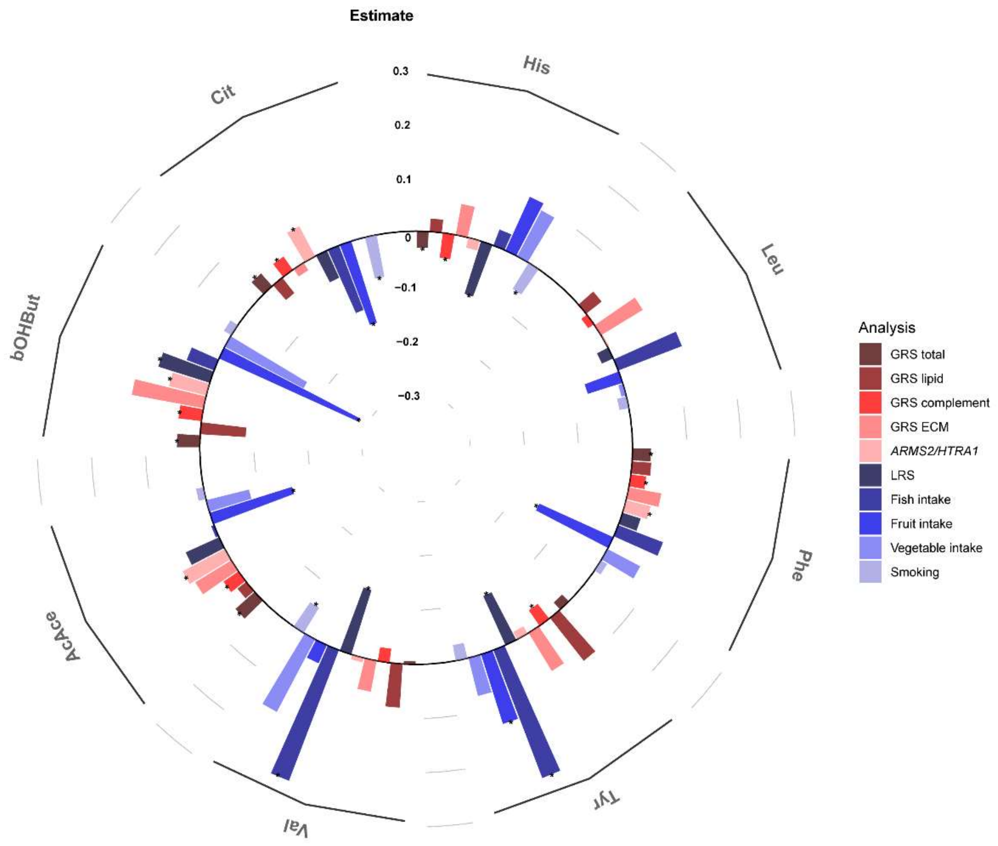

Figure S4. Associations between genetic factors, lifestyle factors, and metabolite levels for late stage AMD. FDR-significant associations from linear regression analyses, adjusted for age, sex, and study site were shown with asterisk. The black circle shows beta estimate zero for the association results. The bars go inwards for the beta estimates <0, and outwards for the beta estimates >0. Genetic risk scores are shown with the gradient of red, while lifestyle risks are shown with the gradient of blue.

Abbreviations: GRS = genetic risk score, LRS = lifestyle risk score. **ARMS2/HTRA1* risk genotype

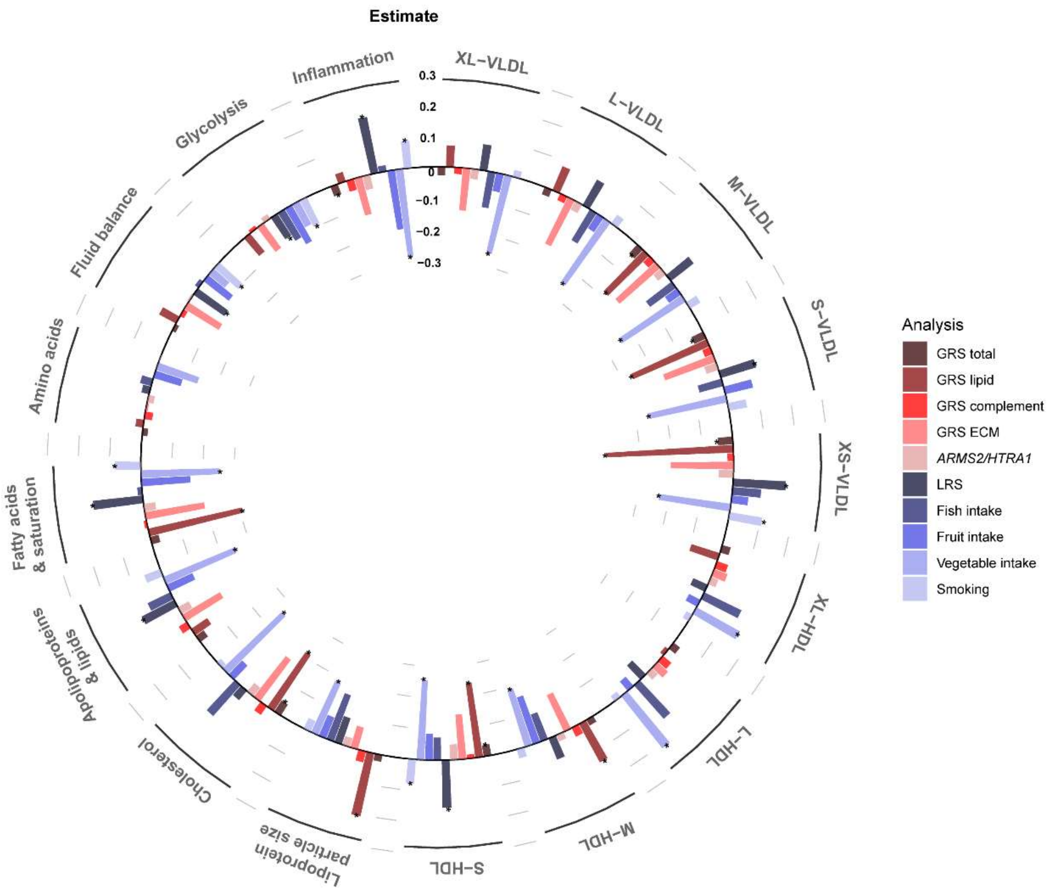

Figure S5. Associations between genetic factors, lifestyle factors, and metabolite levels (per metabolite group) for early- intermediate stage AMD. FDR-significant associations from linear regression analyses, adjusted for age, sex, and study site were shown with asterisk. The black circle shows beta estimate zero for the association results. The bars go inwards for the beta estimates <0, and outwards for the beta estimates >0. Genetic risk scores are shown with the gradient of red, while lifestyle risks are shown with the gradient of blue. Abbreviations: GRS = genetic risk score, LRS = lifestyle risk score.
**ARMS2/HTRA1* risk genotype

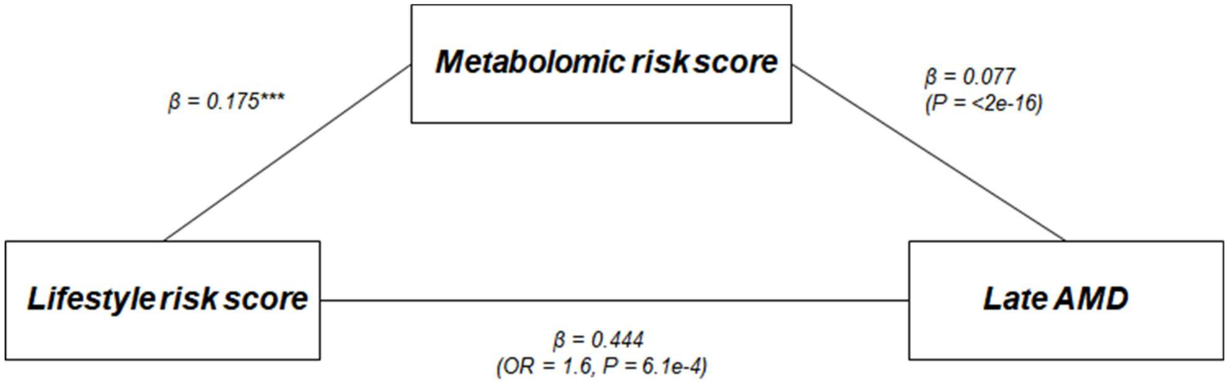

Figure S6. Metabolomic risk score (MRS) as a mediator in the association between lifestyle risk and late AMD. Of the effect of the LRS on late AMD, 19.5% (95%CI 7.0 – 88.0) was mediated by the MRS, after correction for GRS. ***p-value < 0.001.

Table S1. Associations between metabolites and early and intermediate AMD

See excel supplemental. Green highlighted cells show significant association in both pooled and meta-analysis.

AMD patients and controls were age- and sex matched to test metabolite associations in a logistic regression model. The results shown are fixed estimates from our meta-analysis, ordered according to FDR-significance. Abbreviations: AMD = age-related macular degeneration; OR = odds ratio; CI = confidence interval; FDR = false discovery rate.

Table S2. Associations between metabolites and late AMD

See excel supplemental. Green highlighted cells show significant association in both pooled and meta-analysis.

AMD patients and controls were age- and sex matched to test metabolite associations in a logistic regression model. The results shown are fixed estimates from our meta-analysis, ordered according to FDR-significance. Abbreviations: AMD = age-related macular degeneration; OR = odds ratio; CI = confidence interval; FDR = false discovery rate.

Table S3. Associations between metabolites and late AMD (as compared to early- intermediate AMD)

See excel supplemental. Green highlighted cells show significant association in both pooled and meta-analysis.

AMD patients and controls were age- and sex matched to test metabolite associations in a logistic regression model. The results shown are fixed estimates from our meta-analysis, ordered according to FDR-significance. Abbreviations: AMD = age-related macular degeneration; OR = odds ratio; CI = confidence interval; FDR = false discovery rate.

Table S4. Baseline characteristics per study site

	**Subjects in analysis*** (n = 5923)				
	**Controls** (n = 3850)	**Late AMD** (n = 959)	**Early and intermediate AMD** (n = 1114)	*P-value Late AMD*	*P-value Early and intermediate AMD*
**Rotterdam study**	n = 2162	n = 68	n = 395		
Baseline age (mean ± SD)	74.9 ± 5.8	81.5 ± 6.4	77.5 ± 6.5	<0.0001	<0.0001
Sex (% male)	42.2	36.8	43.5	0.369	0.187
BMI (mean ± SD)	26.2 ± 3.5	26.2 ± 3.1	25.9 ± 3.3	0.381	0.069
Current smoking	20.0	32.4	19.7	<0.0001	0.447
Hypertension	45.3	45.6	46.8	0.298	0.651
Diabetes	10.9	16.9	9.3	0.442	0.163
Glucose level					
**EUGENDA-Nijmegen**	n = 785	n = 605	n = 379		
Baseline age (mean ± SD)	68.5 ± 7.7	77.2 ± 7.6	71.8 ± 7.5	<0.0001	<0.0001
Sex (% male)	41.1	41.2	37.8	0.960	0.804
BMI (mean ± SD)	25.7 ± 3.4	26.3 ± 3.9	25.0 ± 3.7	0.006	0.018
Current smoking	5.4	14.7	8.6	<0.0001	0.050
Hypertension	31.3	22.9	26.3	0.034	0.116
Diabetes	6.8	13.2	9.0	0.001	0.252
**EUGENDA-Cologne**	n = 903	n = 286	n = 340		
Baseline age (mean ± SD)	69.9 ± 7.6	77.7 ± 9.5	74.5 ± 9.2	<0.0001	<0.0001
Sex (% male)	41.3	43.0	37.6	0.617	0.099
BMI (mean ± SD)	25.7 ± 3.9	25.6 ± 3.7	25.2 ± 3.4	0.268	0.229
Current smoking	8.3	11.7	5.2	<0.0001	0.249
Hypertension	47.6	52.4	49.1	0.950	0.715
Diabetes	9.2	11.9	9.5	0.445	0.673

*Subjects who had data on routine serum measurements and AMD phenotype. P-values based on regression analyses adjusted for age and sex. Abbreviations: AMD = age-related macular degeneration; BMI = body mass index.

Table S5. Baseline characteristics for subjects in incidence and progression analyses.

	Subjects in incidence and progression analyses*					
	**Incident early-intermediate AMD** (RS only) (n = 374/363)	**Incident late AMD** (RS only) (n = 88/629)	**Incident progression AMD** (RS+EUGENDA) (n = 89/182)	*P-value early-intermediate AMD*	*P-value late AMD*	*P-value progression AMD*
Baseline age (mean ± SD)	73.4 ± 4.9 / 70.7 ± 3.6	74.9 ± 3.8 / 71.2 ± 4.8	74.2 ± 6.8 / 70.6 ± 5.0	<0.001	<0.001	<0.001
Follow-up time (mean ± SD)	8.6 ± 2.5 / 11.4 ± 0.6	9.5 ± 2.4 / 11.5 ± 0.6	4.4 ± 2.3 / 5.8 ± 0.8	<0.001	<0.001	<0.001
Sex (% male)	39.0 / 45.2	53.4 / 41.5	44.9 / 41.2	0.205	0.842	0.903
BMI						
mean ± SD	25.8 ± 3.5 / 25.9 ± 3.1	25.6 ± 3.2 / 25.8 ± 3.3	25.7 ± 3.3 / 25.0 ± 3.1	0.377	0.438	0.057
Smoking			n = 88 / 179			
Former	47.9 / 53.2	53.4 / 51.5	48.9 / 52.0	0.379	0.347	0.868
Current	18.2 / 16.3	14.8 / 15.9	14.8 / 6.7	0.511	0.971	0.037
Hypertension						
% yes	36.9 / 34.7	38.6 / 35.3	39.3 / 38.5	0.970	0.968	0.899

Diabetes type II	n = 354 / 357	n = 87 / 606	n = 88 / 180			
% yes	10.1 / 5.4	5.7 / 7.1	5.7 / 5.0	0.017	0.889	0.915

*Subjects who had data on serum measurements and AMD phenotype.
P-values based on regression analyses adjusted for age and sex. Abbreviations: AMD = age-related macular degeneration; BMI = body mass index; SD = Standard deviation. N = cases/controls.

Table S6. Associations between metabolites and late AMD (incidence analyses)

		**Late** AMD (n = 88)			
**Determinant**	**Determinant type**	**HR**	**95%CI**	**P-value**	**FDR-adjusted P-value**
Cit	Glycolysis	0.71	0.49 - 1.02	0.064900	0.286133
His	Amino Acids	0.86	0.69 - 1.08	0.199640	0.347504
Leu	Amino Acids	0.64	0.38 - 1.09	0.100890	0.286133
Val	Amino Acids	0.82	0.60 - 1.12	0.217190	0.347504
Phe	Amino Acids	0.63	0.36 - 1.10	0.107300	0.286133
Tyr	Amino Acids	0.86	0.63 - 1.18	0.359800	0.479733
AcAce	Keton bodies	0.91	0.72 - 1.16	0.466810	0.533497
bOHBut	Keton bodies	1.02	0.82 - 1.25	0.890900	0.890900

Rotterdam study data only. Pooled cox hazards regression analyses for late AMD adjusted for age, sex and study site. Abbreviations: HR = hazard ratio; CI = confidence interval; FDR = false discovery rate.

Table S7. Associations between metabolites and early-intermediate AMD (incidence analyses)

		Early and intermediate AMD (n = 374)			
**Determinant**	**Determinant type**	**HR**	**95%CI**	**P-value**	**FDR -adjusted P-value**
XLVLDLC	XL-VLDL	0.89	0.79 - 0.99	0.049100	0.1014
XLVLDLCE	XL-VLDL	0.89	0.79 - 0.99	0.044000	0.1014
XLVLDLTG	XL-VLDL	0.88	0.78 - 1.00	0.060700	0.1014
LVLDLP	L-VLDL	0.89	0.79 - 1.01	0.073500	0.1047375
LVLDLL	L-VLDL	0.89	0.79 - 1.01	0.071900	0.1047375
LVLDLPL	L-VLDL	0.90	0.80 - 1.02	0.090300	0.116979545
LVLDLC	L-VLDL	0.89	0.79 - 1.00	0.056800	0.1014
LVLDLCE	L-VLDL	0.90	0.80 - 1.00	0.057300	0.1014
LVLDLFC	L-VLDL	0.89	0.79 - 1.00	0.060600	0.1014
LVLDLTG	L-VLDL	0.89	0.79 - 1.01	0.076300	0.10607561
MVLDLP	M-VLDL	0.89	0.79 - 1.00	0.050500	0.1014
MVLDLL	M-VLDL	0.89	0.79 - 1.0	0.050200	0.1014
MVLDLPL	M-VLDL	0.89	0.79 - 1.00	0.050900	0.1014
MVLDLC	M-VLDL	0.90	0.80 - 1.00	0.055400	0.1014
MVLDLCE	M-VLDL	0.91	0.82 - 1.00	0.066100	0.1014
MVLDLFC	M-VLDL	0.89	0.79 - 1.00	0.054600	0.1014
MVLDLTG	M-VLDL	0.89	0.79 - 1.00	0.054400	0.1014
SVLDLP	S-VLDL	0.89	0.79 - 0.99	0.042500	0.1014
SVLDLL	S-VLDL	0.89	0.79 - 0.99	0.038500	0.1014
SVLDLPL	S-VLDL	0.90	0.81 - 1.01	0.066700	0.1014
SVLDLC	S-VLDL	0.87	0.77 - 0.99	0.035200	0.1014
SVLDLCE	S-VLDL	0.89	0.78 - 1.00	0.058200	0.1014
SVLDLFC	S-VLDL	0.87	0.77 - 0.98	0.025100	0.1014
SVLDLTG	S-VLDL	0.90	0.80 - 1.01	0.067600	0.1014
XSVLDLTG	XS-VLDL	0.93	0.83 - 1.03	0.148000	0.179489362
XLHDLP	XL-HDL	1.17	1.03 - 1.34	0.016800	0.1014
XLHDLL	XL-HDL	1.17	1.03 - 1.33	0.018800	0.1014
XLHDLPL	XL-HDL	1.20	1.05 - 1.37	0.008730	0.0855
XLHDLC	XL-HDL	1.13	1.00 - 1.28	0.056600	0.1014
XLHDLCE	XL-HDL	1.13	0.99 - 1.28	0.060800	0.1014
XLHDLFC	XL-HDL	1.13	0.99 - 1.28	0.064500	0.1014
LHDLP	L-HDL	1.20	1.04 - 1.37	0.010200	0.0855
LHDLL	L-HDL	1.19	1.04 - 1.36	0.010500	0.0855
LHDLPL	L-HDL	1.17	1.03 - 1.35	0.020000	0.1014
LHDLC	L-HDL	1.20	1.05 - 1.36	0.007890	0.0855
LHDLCE	L-HDL	1.21	1.06 - 1.38	0.005290	0.0855
LHDLFC	L-HDL	1.15	1.02 - 1.30	0.026600	0.1014

MHDLL	M-HDL	1.09	0.94 - 1.27	0.238000	0.27132
MHDLPL	M-HDL	1.08	0.94 - 1.25	0.278000	0.304730769
MHDLFC	M-HDL	1.05	0.93 - 1.19	0.404000	0.426444444
MHDLTG	M-HDL	0.93	0.84 - 1.03	0.140000	0.173478261
SHDLTG	S-HDL	0.91	0.82 - 1.02	0.095000	0.120333333
VLDLD	Lipoproteint particle size	0.89	0.79 - 1.01	0.080200	0.10684186
HDLD	Lipoproteint particle size	1.19	1.05 - 1.35	0.006630	0.0855
VLDLC	Cholesterol	0.88	0.79 - 0.99	0.035500	0.1014
HDLC	Cholesterol	1.17	1.02 - 1.34	0.021300	0.1014
HDL2C	Cholesterol	1.16	1.02 - 1.32	0.024900	0.1014
SerumTG	Apolipoproteins & lipids	0.90	0.80 - 1.01	0.080600	0.10684186
VLDLTG	Fatty acids & saturation	0.89	0.79 - 1.01	0.060600	0.1014
HDLTG	Apolipoproteins & lipids	0.96	0.87 - 1.06	0.452000	0.468436364
ApoA1	Apolipoproteins & lipids	1.10	0.96 - 1.26	0.168000	0.195428571
TotFA	Fatty acids & saturation	0.92	0.81 - 1.04	0.164000	0.19475
MUFA	Fatty acids & saturation	0.93	0.82 - 1.05	0.258000	0.288352941
Cit	Glycolysis	0.96	0.81 - 1.14	0.636000	0.647357143
Ile	Amino acids	0.78	0.67 - 0.91	0.001200	0.0684
Alb	Fluid balance	0.98	0.86 - 1.11	0.793000	0.793
Gp	Inflammation	0.94	0.82 - 1.08	0.391000	0.420509434

Rotterdam study data only. Pooled cox hazards regression analyses for early and intermediate AMD adjusted for age, sex and study site. Abbreviations: HR = hazard ratio; CI = confidence interval; FDR = false discovery rate.

Table S8. Pathway analysis significant metabolites late AMD

Pathways	Total	Expected	Hits	Raw P-value	FDR P-value	Impact
Phenylalanine, tyrosine and tryptophan biosynthesis	4	0.020645	2	0.00013922	0.0058473	1
Synthesis and degradation of ketone bodies	5	0.025806	2	0.00023144	0.0064802	0.6
Phenylalanine metabolism	10	0.051613	2	0.0010281	0.014393	0.35714
Butanoate metabolism	15	0.077419	2	0.002368	0.028415	0.11111
Aminoacyl-tRNA biosynthesis	48	0.24774	5	1,21E-02	0.00010135	0
Valine, leucine and isoleucine biosynthesis	8	0.04129	2	0.00064301	0.013503	0
Valine, leucine and isoleucine degradation	40	0.20645	3	0.00081601	0.013709	0
Tyrosine metabolism	42	0.21677	2	0.018102	0.19007	0.13972
Ubiquinone and other terpenoid-quinone biosynthesis	9	0.046452	1	0.04562	0.42578	0
Histidine metabolism	16	0.082581	1	0.079832	0.66946	0.22131
Citrate cycle (TCA cycle)	20	0.10323	1	0.098896	0.66946	0.09038
Pantothenate and CoA biosynthesis	19	0.098065	1	0.094163	0.66946	0
beta-Alanine metabolism	21	0.10839	1	0.10361	0.66946	0
Alanine, aspartate and glutamate metabolism	28	0.14452	1	0.13599	0.81594	0
Glyoxylate and dicarboxylate metabolism	32	0.16516	1	0.15403	0.86257	0.03175

Abbreviations: FDR = false discovery rate.

Table S9. Association of fasting serum glucose and diabetes with AMD stage

	**No AMD** (n = 2018)	**Early-Intermediate AMD** (n = 378)	**Late AMD** (n = 59)	**P-value**	**OR** (95% CI) **Early-Intermediate AMD**	**P-value**	**OR** (95% CI) **Late AMD**	**P-value**
Fasting serum glucose (mean ± SD)	5.9 ± 1.4	5.8 ± 1.4	5.9 ± 1.1	0.830	1.0 (0.9 – 1.1)	0.537	OR 1.0 (0.8 – 1.2)	0.845
Diabetes mellitus 2 (% yes)	32.7	31.5	35.7	0.809	1.0 (0.7 – 1.2)	0. 712	OR 1.2 (0.7 – 2.2)	0.458

Abbreviations: SD = standard deviation; AMD = age-related macular degeneration; OR = odds ratio; CI = confidence interval

Table S10. Associations between GRS, LRS and metabolites (early and intermediate AMD)

**Determinant**	**Outcome**	**Outcome type**	**Estimate**	**SE**	**P-value**	**FDR-adjusted**

						P-value adjust
**GRS total**	XLVLDLC	XL-VLDL	-0.037	0.017	8.08E-80	4.93026E-79
	XLVLDLCE	XL-VLDL	-0.040	0.017	0.485964955	0.502438343
	XLVLDLTG	XL-VLDL	-0.028	0.017	0.001974051	0.003541679
	LVLDLP	L-VLDL	-0.030	0.017	0.047911716	0.066423061
	LVLDLL	L-VLDL	-0.030	0.017	1.08E-29	4.70552E-29
	LVLDLPL	L-VLDL	-0.030	0.017	4.37E-156	8.8951E-155
	LVLDLC	L-VLDL	-0.035	0.017	1.11E-151	1.6851E-150
	LVLDLCE	L-VLDL	-0.039	0.017	0.123065788	0.141641756
	LVLDLFC	L-VLDL	-0.031	0.017	0.045731982	0.064875602
	LVLDLTG	L-VLDL	-0.028	0.017	2.57E-24	1.04676E-23
	MVLDLP	M-VLDL	-0.033	0.017	7.56E-166	2.3047E-164
	MVLDLL	M-VLDL	-0.034	0.017	1.37E-173	8.3364E-172
	MVLDLPL	M-VLDL	-0.034	0.017	0.000588778	0.001197181
	MVLDLC	M-VLDL	-0.043	0.017	0.071389094	0.087094694
	MVLDLCE	M-VLDL	-0.048	0.017	2.73E-11	7.92487E-11
	MVLDLFC	M-VLDL	-0.036	0.017	1.39E-143	1.6943E-142
	MVLDLTG	M-VLDL	-0.030	0.017	0.293792252	0.308988403
	SVLDLP	S-VLDL	-0.048	0.016	0.278465838	0.303328859
	SVLDLL	S-VLDL	-0.050	0.016	0.058206738	0.075544915
	SVLDLPL	S-VLDL	-0.054	0.016	8.72E-07	2.21627E-06
	SVLDLC	S-VLDL	-0.053	0.014	1.33E-30	6.2498E-30
	SVLDLCE	S-VLDL	-0.051	0.014	1.86E-40	1.03331E-39
	SVLDLFC	S-VLDL	-0.052	0.015	0.240530602	0.266770304
	SVLDLTG	S-VLDL	-0.036	0.017	0.01076987	0.018248946
	XSVLDLTG	XS-VLDL	-0.055	0.017	0.873017392	0.873017392
	XLHDLP	XL-HDL	0.028	0.016	0.813321945	0.82687731
	XLHDLL	XL-HDL	0.028	0.016	3.10E-09	8.22544E-09
	XLHDLPL	XL-HDL	0.036	0.016	0.019178608	0.030786713
	XLHDLC	XL-HDL	0.019	0.016	0.288678101	0.308936213
	XLHDLCE	XL-HDL	0.015	0.016	1.45E-05	3.41133E-05
	XLHDLFC	XL-HDL	0.026	0.016	2.01E-10	5.58691E-10
	LHDLP	L-HDL	0.032	0.016	2.20E-39	1.11626E-38
	LHDLL	L-HDL	0.032	0.016	8.80E-12	2.98262E-11
	LHDLPL	L-HDL	0.034	0.015	0.079144908	0.094663517
	LHDLC	L-HDL	0.033	0.016	1.23E-06	3.00528E-06
	LHDLCE	L-HDL	0.032	0.016	3.95E-84	2.67717E-83
	LHDLFC	L-HDL	0.034	0.016	0.067181088	0.085375966
	MHDLL	M-HDL	0.027	0.014	1.93E-11	5.87401E-11
	MHDLPL	M-HDL	0.027	0.014	0.000639978	0.001259311
	MHDLFC	M-HDL	0.028	0.015	0.000355019	0.000773434

	MHDLTG	M-HDL	-0.031	0.016	1.49E-11	4.7968E-11
	SHDLTG	S-HDL	-0.043	0.017	6.49E-111	4.9509E-110
	VLDLD	Lipoproteint particle size	-0.017	0.016	0.018908036	0.030786713
	HDLD	Lipoproteint particle size	0.028	0.016	0.022203178	0.033859847
	VLDLC	Cholesterol	-0.054	0.016	0.050742942	0.068784877
	HDLC	Cholesterol	0.036	0.016	5.78E-127	5.8792E-126
	HDL2C	Cholesterol	0.041	0.016	4.79E-22	1.82729E-21
	SerumTG	Apolipoproteins & lipids	-0.038	0.017	0.021941102	0.033859847
	VLDLTG	Apolipoproteins & lipids	-0.033	0.017	0.009234056	0.01609364
	HDLTG	Apolipoproteins & lipids	-0.037	0.017	0.08128294	0.095351142
	ApoA1	Apolipoproteins & lipids	0.023	0.015	1.24E-114	1.0841E-113
	TotFA	Fatty acids & saturation	-0.037	0.016	2.49E-19	8.92732E-19
	MUFA	Fatty acids & saturation	-0.027	0.015	0.023377965	0.03478185
	Cit	Glycolysis	0.038	0.011	0.000541	0.001442
	Ile	Amino acids	-0.019	0.015	0.001169968	0.002230251
	Alb	Fluid balance	-0.016	0.015	0.070705677	0.087094694
	Gp	Inflammation	-0.039	0.017	0.001293426	0.002390878
	XLVLDLP	XL-VLDL	-0.031	0.017	0.00049546	0.001042175
	XLVLDLL	XL-VLDL	-0.031	0.017	0.052346581	0.069416118
	XLVLDLFC	XL-VLDL	-0.033	0.017	4.67E-05	0.000105473
	XSVLDLP	XS-VLDL	-0.057	0.016	0.15196811	0.17166768
**GRS complement**	XLVLDLC	XL-VLDL	-0.021	0.016	0.194423341	0.257203344
	XLVLDLCE	XL-VLDL	-0.023	0.016	0.165688875	0.257203344
	XLVLDLTG	XL-VLDL	-0.020	0.016	0.203874323	0.257203344
	LVLDLP	L-VLDL	-0.022	0.016	0.16375708	0.257203344
	LVLDLL	L-VLDL	-0.023	0.016	0.16275467	0.257203344
	LVLDLPL	L-VLDL	-0.023	0.016	0.153565314	0.257203344
	LVLDLC	L-VLDL	-0.021	0.016	0.187628794	0.257203344
	LVLDLCE	L-VLDL	-0.021	0.016	0.194479401	0.257203344
	LVLDLFC	L-VLDL	-0.022	0.016	0.171141313	0.257203344
	LVLDLTG	L-VLDL	-0.022	0.016	0.166617716	0.257203344
	MVLDLP	M-VLDL	-0.024	0.016	0.144463087	0.257203344
	MVLDLL	M-VLDL	-0.023	0.016	0.148338327	0.257203344
	MVLDLPL	M-VLDL	-0.023	0.016	0.163159055	0.257203344
	MVLDLC	M-VLDL	-0.021	0.016	0.199112538	0.257203344
	MVLDLCE	M-VLDL	-0.019	0.016	0.229037958	0.273947362
	MVLDLFC	M-VLDL	-0.021	0.016	0.188942972	0.257203344
	MVLDLTG	M-VLDL	-0.024	0.016	0.133012045	0.257203344
	SVLDLP	S-VLDL	-0.023	0.016	0.142542822	0.257203344
	SVLDLL	S-VLDL	-0.023	0.015	0.134465386	0.257203344
	SVLDLPL	S-VLDL	-0.025	0.016	0.109713987	0.257203344

	SVLDLC	S-VLDL	-0.020	0.013	0.131020019	0.257203344
	SVLDLCE	S-VLDL	-0.018	0.013	0.170193725	0.257203344
	SVLDLFC	S-VLDL	-0.022	0.014	0.122901179	0.257203344
	SVLDLTG	S-VLDL	-0.021	0.016	0.20667586	0.257203344
	XSVLDLTG	XS-VLDL	-0.012	0.016	0.461865791	0.521737283
	XLHDLP	XL-HDL	0.036	0.015	0.018135232	0.156814577
	XLHDLL	XL-HDL	0.036	0.015	0.019925658	0.156814577
	XLHDLPL	XL-HDL	0.042	0.015	0.004813395	0.156814577
	XLHDLC	XL-HDL	0.026	0.016	0.096786431	0.257203344
	XLHDLCE	XL-HDL	0.022	0.016	0.162200471	0.257203344
	XLHDLFC	XL-HDL	0.035	0.015	0.025000522	0.156814577
	LHDLP	L-HDL	0.034	0.015	0.025175455	0.156814577
	LHDLL	L-HDL	0.034	0.015	0.025707308	0.156814577
	LHDLPL	L-HDL	0.035	0.015	0.019176473	0.156814577
	LHDLC	L-HDL	0.033	0.015	0.0348315	0.177060125
	LHDLCE	L-HDL	0.032	0.015	0.038137831	0.178198158
	LHDLFC	L-HDL	0.034	0.016	0.028536818	0.158249629
	MHDLL	M-HDL	0.021	0.013	0.118315417	0.257203344
	MHDLPL	M-HDL	0.024	0.013	0.070497486	0.252961568
	MHDLFC	M-HDL	0.030	0.015	0.040897938	0.178198158
	MHDLTG	M-HDL	-0.003	0.016	0.86120415	0.875557552
	SHDLTG	S-HDL	-0.009	0.016	0.574629929	0.604352166
	VLDLD	Lipoproteint particle size	-0.026	0.016	0.095366636	0.257203344
	HDLD	Lipoproteint particle size	0.035	0.016	0.023681163	0.156814577
	VLDLC	Cholesterol	-0.022	0.015	0.149130462	0.257203344
	HDLC	Cholesterol	0.034	0.015	0.02241911	0.156814577
	HDL2C	Cholesterol	0.035	0.015	0.01892046	0.156814577
	SerumTG	Apolipoproteins & lipids	-0.017	0.016	0.297404174	0.348877973
	VLDLTG	Apolipoproteins & lipids	-0.022	0.016	0.167506154	0.257203344
	HDLTG	Apolipoproteins & lipids	0.008	0.016	0.62364376	0.644784227
	ApoA1	Apolipoproteins & lipids	0.028	0.015	0.053919207	0.206491028
	TotFA	Fatty acids & saturation	-0.009	0.015	0.550159866	0.599281282
	MUFA	Fatty acids & saturation	0.000	0.015	0.977381388	0.977381388
	Cit	Glycolysis	0.027	0.011	0.015141	0.027325
	Ile	Amino acids	-0.025	0.015	0.092872809	0.257203344
	Alb	Fluid balance	-0.014	0.014	0.337015757	0.387886061
	Gp	Inflammation	-0.031	0.016	0.054161581	0.206491028
	XLVLDLP	XL-VLDL	-0.022	0.016	0.178840823	0.257203344
	XLVLDLL	XL-VLDL	-0.022	0.016	0.178633399	0.257203344
	XLVLDLFC	XL-VLDL	-0.020	0.016	0.210822413	0.257203344
	XSVLDLP	XS-VLDL	-0.009	0.015	0.52942213	0.587177271

**GRS lipid**	XLVLDLC	XL-VLDL	-0.004	0.016	0.820501551	0.932531803
	XLVLDLCE	XL-VLDL	-0.010	0.016	0.548873295	0.779107934
	XLVLDLTG	XL-VLDL	0.017	0.016	0.279157921	0.567621105
	LVLDLP	L-VLDL	0.016	0.016	0.319767178	0.598876093
	LVLDLL	L-VLDL	0.015	0.016	0.364491671	0.653940939
	LVLDLPL	L-VLDL	0.014	0.016	0.384781933	0.670619941
	LVLDLC	L-VLDL	-0.002	0.016	0.889375114	0.932531803
	LVLDLCE	L-VLDL	-0.013	0.016	0.418396271	0.686226181
	LVLDLFC	L-VLDL	0.008	0.016	0.619216662	0.821135139
	LVLDLTG	L-VLDL	0.021	0.016	0.193300734	0.436716474
	MVLDLP	M-VLDL	0.009	0.016	0.564081558	0.78202216
	MVLDLL	M-VLDL	0.006	0.016	0.701853467	0.891938781
	MVLDLPL	M-VLDL	0.002	0.016	0.92403007	0.932531803
	MVLDLC	M-VLDL	-0.026	0.016	0.109321858	0.317553969
	MVLDLCE	M-VLDL	-0.047	0.016	0.003610801	0.018354904
	MVLDLFC	M-VLDL	-0.002	0.016	0.923397512	0.932531803
	MVLDLTG	M-VLDL	0.022	0.016	0.176367027	0.429107291
	SVLDLP	S-VLDL	-0.035	0.016	0.029156185	0.104619253
	SVLDLL	S-VLDL	-0.041	0.015	0.009268543	0.03665599
	SVLDLPL	S-VLDL	-0.045	0.016	0.004659515	0.02186388
	SVLDLC	S-VLDL	-0.070	0.013	2.06E-07	3.13495E-06
	SVLDLCE	S-VLDL	-0.076	0.013	9.96E-09	3.03635E-07
	SVLDLFC	S-VLDL	-0.055	0.014	0.000145502	0.00110945
	SVLDLTG	S-VLDL	-0.005	0.016	0.740117232	0.909340956
	XSVLDLTG	XS-VLDL	-0.076	0.016	2.59E-06	3.15879E-05
	XLHDLP	XL-HDL	-0.021	0.015	0.170291256	0.429107291
	XLHDLL	XL-HDL	-0.021	0.015	0.18289819	0.429107291
	XLHDLPL	XL-HDL	-0.012	0.015	0.427485162	0.686226181
	XLHDLC	XL-HDL	-0.024	0.016	0.125020701	0.336964936
	XLHDLCE	XL-HDL	-0.027	0.016	0.087141673	0.265782102
	XLHDLFC	XL-HDL	-0.016	0.015	0.299573284	0.589482914
	LHDLP	L-HDL	-0.003	0.015	0.861057089	0.932531803
	LHDLL	L-HDL	-0.001	0.015	0.932531803	0.932531803
	LHDLPL	L-HDL	0.002	0.015	0.904326999	0.932531803
	LHDLC	L-HDL	0.003	0.015	0.845316966	0.932531803
	LHDLCE	L-HDL	0.004	0.015	0.818706551	0.932531803
	LHDLFC	L-HDL	0.001	0.016	0.928049173	0.932531803
	MHDLL	M-HDL	0.040	0.013	0.002879089	0.015965859
	MHDLPL	M-HDL	0.034	0.013	0.008839197	0.03665599
	MHDLFC	M-HDL	0.007	0.015	0.650894833	0.844778401
	MHDLTG	M-HDL	-0.041	0.016	0.009614686	0.03665599

	SHDLTG	S-HDL	-0.069	0.016	2.89E-05	0.000294216
	VLDLD	Lipoproteint particle size	0.058	0.016	0.00026024	0.001763851
	HDLD	Lipoproteint particle size	-0.019	0.016	0.221510321	0.482576056
	VLDLC	Cholesterol	-0.059	0.015	0.000107002	0.000932448
	HDLC	Cholesterol	0.008	0.015	0.606641973	0.821135139
	HDL2C	Cholesterol	0.023	0.015	0.127052353	0.336964936
	SerumTG	Apolipoproteins & lipids	-0.016	0.016	0.323982149	0.598876093
	VLDLTG	Apolipoproteins & lipids	0.010	0.016	0.535155173	0.779107934
	HDLTG	Apolipoproteins & lipids	-0.087	0.016	5.76E-08	1.17192E-06
	ApoA1	Apolipoproteins & lipids	0.005	0.015	0.745361439	0.909340956
	TotFA	Fatty acids & saturation	-0.055	0.015	0.000326243	0.001990083
	MUFA	Fatty acids & saturation	-0.030	0.015	0.041452275	0.140477155
	Cit	Glycolysis	-0.011	0.011	0.335904	0.537446
	Ile	Amino acids	0.009	0.015	0.549207232	0.779107934
	Alb	Fluid balance	0.017	0.014	0.237442928	0.499448917
	Gp	Inflammation	0.011	0.016	0.492592669	0.75120382
	XLVLDLP	XL-VLDL	0.013	0.016	0.410742636	0.686226181
	XLVLDLL	XL-VLDL	0.012	0.016	0.457572973	0.715691061
	XLVLDLFC	XL-VLDL	0.003	0.016	0.84772581	0.932531803
	XSVLDLP	XS-VLDL	-0.115	0.015	1.75E-14	1.06593E-12
**GRS ECM**	XLVLDLC	XL-VLDL	-0.035	0.016	0.027976739	0.063523996
	XLVLDLCE	XL-VLDL	-0.034	0.016	0.034769697	0.064271257
	XLVLDLTG	XL-VLDL	-0.034	0.016	0.033875387	0.064271257
	LVLDLP	L-VLDL	-0.035	0.016	0.027919426	0.063523996
	LVLDLL	L-VLDL	-0.035	0.016	0.02625642	0.063523996
	LVLDLPL	L-VLDL	-0.037	0.016	0.021334956	0.063523996
	LVLDLC	L-VLDL	-0.036	0.016	0.024693007	0.063523996
	LVLDLCE	L-VLDL	-0.035	0.016	0.029783999	0.063523996
	LVLDLFC	L-VLDL	-0.038	0.016	0.018283707	0.063523996
	LVLDLTG	L-VLDL	-0.034	0.016	0.032282687	0.063523996
	MVLDLP	M-VLDL	-0.034	0.016	0.031564851	0.063523996
	MVLDLL	M-VLDL	-0.035	0.016	0.02952692	0.063523996
	MVLDLPL	M-VLDL	-0.035	0.016	0.028549058	0.063523996
	MVLDLC	M-VLDL	-0.038	0.016	0.017794291	0.063523996
	MVLDLCE	M-VLDL	-0.037	0.016	0.017909391	0.063523996
	MVLDLFC	M-VLDL	-0.036	0.016	0.023565408	0.063523996
	MVLDLTG	M-VLDL	-0.032	0.016	0.041718506	0.072709396
	SVLDLP	S-VLDL	-0.037	0.016	0.016872997	0.063523996
	SVLDLL	S-VLDL	-0.037	0.015	0.015107203	0.063523996
	SVLDLPL	S-VLDL	-0.040	0.016	0.01063438	0.063523996
	SVLDLC	S-VLDL	-0.032	0.013	0.015839166	0.063523996

	SVLDLCE	S-VLDL	-0.028	0.013	0.029606086	0.063523996
	SVLDLFC	S-VLDL	-0.035	0.014	0.01292943	0.063523996
	SVLDLTG	S-VLDL	-0.034	0.016	0.035881944	0.06437643
	XSVLDLTG	XS-VLDL	-0.043	0.016	0.006762943	0.063523996
	XLHDLP	XL-HDL	0.007	0.015	0.654268873	0.700182479
	XLHDLL	XL-HDL	0.007	0.015	0.63787845	0.700182479
	XLHDLPL	XL-HDL	0.007	0.015	0.635413956	0.700182479
	XLHDLC	XL-HDL	0.009	0.015	0.545147685	0.689629211
	XLHDLCE	XL-HDL	0.011	0.015	0.490456099	0.664840489
	XLHDLFC	XL-HDL	0.006	0.015	0.710098134	0.746827348
	LHDLP	L-HDL	0.007	0.015	0.646136045	0.700182479
	LHDLL	L-HDL	0.007	0.015	0.633244557	0.700182479
	LHDLPL	L-HDL	0.007	0.015	0.645838834	0.700182479
	LHDLC	L-HDL	0.009	0.015	0.572183535	0.698063912
	LHDLCE	L-HDL	0.009	0.015	0.553964448	0.689629211
	LHDLFC	L-HDL	0.007	0.015	0.635293758	0.700182479
	MHDLL	M-HDL	-0.011	0.013	0.379551243	0.526196042
	MHDLPL	M-HDL	-0.008	0.013	0.531924551	0.689629211
	MHDLFC	M-HDL	-0.019	0.015	0.207293856	0.30841281
	MHDLTG	M-HDL	-0.028	0.015	0.071827074	0.115301356
	SHDLTG	S-HDL	-0.031	0.016	0.055181595	0.090975062
	VLDLD	Lipoproteint particle size	-0.016	0.016	0.291081961	0.422761895
	HDLD	Lipoproteint particle size	0.010	0.015	0.512503756	0.679624546
	VLDLC	Cholesterol	-0.037	0.015	0.013103121	0.063523996
	HDLC	Cholesterol	0.002	0.015	0.889016483	0.903833425
	HDL2C	Cholesterol	0.004	0.015	0.779016859	0.80542421
	SerumTG	Apolipoproteins & lipids	-0.039	0.016	0.015865664	0.063523996
	VLDLTG	Apolipoproteins & lipids	-0.035	0.016	0.028307986	0.063523996
	HDLTG	Apolipoproteins & lipids	-0.036	0.016	0.020410425	0.063523996
	ApoA1	Apolipoproteins & lipids	-0.014	0.014	0.336050484	0.476722779
	TotFA	Fatty acids & saturation	-0.042	0.015	0.005192295	0.063523996
	MUFA	Fatty acids & saturation	-0.042	0.014	0.004014835	0.063523996
	Cit	Glycolysis	-0.002	0.011	0.819322	0.936368
	Ile	Amino acids	-0.002	0.014	0.913345524	0.913345524
	Alb	Fluid balance	-0.028	0.014	0.044001965	0.074558885
	Gp	Inflammation	-0.027	0.016	0.082229086	0.128614724
	XLVLDLP	XL-VLDL	-0.036	0.016	0.024512932	0.063523996
	XLVLDLL	XL-VLDL	-0.036	0.016	0.024104063	0.063523996
	XLVLDLFC	XL-VLDL	-0.038	0.016	0.017676969	0.063523996
	XSVLDLP	XS-VLDL	-0.032	0.015	0.030546317	0.063523996
ARMS2/HTRA1	XLVLDLC	XL-VLDL	-0.027	0.017	0.107386368	0.331092995

	XLVLDLCE	XL-VLDL	-0.029	0.017	0.0829872	0.331092995
	XLVLDLTG	XL-VLDL	-0.023	0.017	0.158474003	0.331092995
	LVLDLP	L-VLDL	-0.022	0.017	0.180401901	0.331092995
	LVLDLL	L-VLDL	-0.022	0.017	0.178857564	0.331092995
	LVLDLPL	L-VLDL	-0.021	0.017	0.194913251	0.331092995
	LVLDLC	L-VLDL	-0.024	0.017	0.153798184	0.331092995
	LVLDLCE	L-VLDL	-0.028	0.017	0.091471408	0.331092995
	LVLDLFC	L-VLDL	-0.020	0.017	0.223278458	0.340499649
	LVLDLTG	L-VLDL	-0.021	0.017	0.195953621	0.331092995
	MVLDLP	M-VLDL	-0.023	0.017	0.166725367	0.331092995
	MVLDLL	M-VLDL	-0.023	0.017	0.164327264	0.331092995
	MVLDLPL	M-VLDL	-0.020	0.017	0.219614233	0.340499649
	MVLDLC	M-VLDL	-0.025	0.017	0.139919938	0.331092995
	MVLDLCE	M-VLDL	-0.023	0.017	0.157625175	0.331092995
	MVLDLFC	M-VLDL	-0.025	0.017	0.139719966	0.331092995
	MVLDLTG	M-VLDL	-0.023	0.017	0.169212007	0.331092995
	SVLDLP	S-VLDL	-0.027	0.016	0.097036217	0.331092995
	SVLDLL	S-VLDL	-0.027	0.016	0.094304223	0.331092995
	SVLDLPL	S-VLDL	-0.029	0.016	0.078301202	0.331092995
	SVLDLC	S-VLDL	-0.020	0.014	0.144505736	0.331092995
	SVLDLCE	S-VLDL	-0.018	0.014	0.194920211	0.331092995
	SVLDLFC	S-VLDL	-0.024	0.015	0.10551083	0.331092995
	SVLDLTG	S-VLDL	-0.025	0.017	0.132503186	0.331092995
	XSVLDLTG	XS-VLDL	-0.032	0.017	0.052278063	0.331092995
	XLHDLP	XL-HDL	0.012	0.016	0.435224852	0.514572814
	XLHDLL	XL-HDL	0.012	0.016	0.448948094	0.514572814
	XLHDLPL	XL-HDL	0.016	0.015	0.310631656	0.403160234
	XLHDLC	XL-HDL	0.008	0.016	0.630612807	0.651989512
	XLHDLCE	XL-HDL	0.007	0.016	0.646473252	0.657247807
	XLHDLFC	XL-HDL	0.008	0.016	0.604474414	0.635740332
	LHDLP	L-HDL	0.017	0.016	0.273181918	0.378836491
	LHDLL	L-HDL	0.017	0.016	0.279469542	0.378836491
	LHDLPL	L-HDL	0.020	0.015	0.200826898	0.331092995
	LHDLC	L-HDL	0.015	0.016	0.350024735	0.435745078
	LHDLCE	L-HDL	0.015	0.016	0.344371831	0.435745078
	LHDLFC	L-HDL	0.014	0.016	0.375488369	0.45809581
	MHDLL	M-HDL	0.015	0.014	0.275317822	0.378836491
	MHDLPL	M-HDL	0.009	0.013	0.485657289	0.533263822
	MHDLFC	M-HDL	0.017	0.015	0.265815482	0.378836491
	MHDLTG	M-HDL	-0.023	0.016	0.157425411	0.331092995
	SHDLTG	S-HDL	-0.030	0.017	0.074163507	0.331092995

	VLDLD	Lipoproteint particle size	-0.018	0.016	0.25906106	0.378836491
	HDLD	Lipoproteint particle size	0.012	0.016	0.455523475	0.514572814
	VLDLC	Cholesterol	-0.025	0.016	0.112246378	0.331092995
	HDLC	Cholesterol	0.022	0.015	0.145923515	0.331092995
	HDL2C	Cholesterol	0.022	0.016	0.164965294	0.331092995
	SerumTG	Apolipoproteins & lipids	-0.027	0.017	0.102678134	0.331092995
	VLDLTG	Apolipoproteins & lipids	-0.024	0.017	0.142309027	0.331092995
	HDLTG	Apolipoproteins & lipids	-0.024	0.016	0.138961129	0.331092995
	ApoA1	Apolipoproteins & lipids	0.016	0.015	0.303387856	0.402318679
	TotFA	Fatty acids & saturation	-0.012	0.016	0.453693693	0.514572814
	MUFA	Fatty acids & saturation	-0.023	0.015	0.121558653	0.331092995
	Cit	Glycolysis	0.009	0.012	0.489553672	0.533263822
	Ile	Amino acids	-0.009	0.015	0.547745167	0.586183425
	Alb	Fluid balance	0.004	0.015	0.802845414	0.802845414
	Gp	Inflammation	-0.030	0.016	0.065421774	0.331092995
	XLVLDLP	XL-VLDL	-0.025	0.017	0.1282412	0.331092995
	XLVLDLL	XL-VLDL	-0.025	0.017	0.125439176	0.331092995
	XLVLDLFC	XL-VLDL	-0.026	0.017	0.125464843	0.331092995
	XSVLDLP	XS-VLDL	-0.019	0.015	0.212470927	0.340499649
**LRS**	XLVLDLC	XL-VLDL	0.030	0.017	0.086614678	0.14676376
	XLVLDLCE	XL-VLDL	0.030	0.017	0.089126807	0.14693879
	XLVLDLTG	XL-VLDL	0.040	0.017	0.018944624	0.046224882
	LVLDLP	L-VLDL	0.044	0.017	0.01112086	0.037985362
	LVLDLL	L-VLDL	0.043	0.017	0.011831506	0.037985362
	LVLDLPL	L-VLDL	0.042	0.017	0.01425698	0.043483789
	LVLDLC	L-VLDL	0.037	0.017	0.033686509	0.068495903
	LVLDLCE	L-VLDL	0.030	0.017	0.081570552	0.146347166
	LVLDLFC	L-VLDL	0.044	0.017	0.01141108	0.037985362
	LVLDLTG	L-VLDL	0.045	0.017	0.008405464	0.037985362
	MVLDLP	M-VLDL	0.041	0.017	0.016712096	0.046175081
	MVLDLL	M-VLDL	0.041	0.017	0.018167245	0.046175081
	MVLDLPL	M-VLDL	0.043	0.017	0.011784734	0.037985362
	MVLDLC	M-VLDL	0.030	0.017	0.085277649	0.14676376
	MVLDLCE	M-VLDL	0.016	0.017	0.362684793	0.433799458
	MVLDLFC	M-VLDL	0.044	0.017	0.011118565	0.037985362
	MVLDLTG	M-VLDL	0.044	0.017	0.011063645	0.037985362
	SVLDLP	S-VLDL	0.043	0.017	0.010717884	0.037985362
	SVLDLL	S-VLDL	0.040	0.017	0.015864294	0.046081997
	SVLDLPL	S-VLDL	0.051	0.017	0.002372416	0.01808967
	SVLDLC	S-VLDL	0.008	0.015	0.56080093	0.587286474
	SVLDLCE	S-VLDL	-0.007	0.014	0.600218654	0.610222298

	SVLDLFC	S-VLDL	0.037	0.015	0.017675002	0.046175081
	SVLDLTG	S-VLDL	0.053	0.017	0.002191714	0.01808967
	XSVLDLTG	XS-VLDL	0.071	0.017	3.65E-05	0.000742142
	XLHDLP	XL-HDL	-0.017	0.016	0.300628276	0.373440645
	XLHDLL	XL-HDL	-0.016	0.016	0.30609889	0.373440645
	XLHDLPL	XL-HDL	-0.019	0.016	0.231884828	0.307499446
	XLHDLC	XL-HDL	-0.013	0.016	0.409832579	0.462959025
	XLHDLCE	XL-HDL	-0.013	0.016	0.428293288	0.475016192
	XLHDLFC	XL-HDL	-0.014	0.016	0.386958894	0.445367784
	LHDLP	L-HDL	-0.017	0.016	0.276280227	0.351106122
	LHDLL	L-HDL	-0.018	0.016	0.263651436	0.342185906
	LHDLPL	L-HDL	-0.014	0.016	0.38612311	0.445367784
	LHDLC	L-HDL	-0.022	0.016	0.18477416	0.274907897
	LHDLCE	L-HDL	-0.022	0.016	0.179774618	0.274156293
	LHDLFC	L-HDL	-0.021	0.016	0.207775098	0.301768595
	MHDLL	M-HDL	0.018	0.014	0.222761917	0.307499446
	MHDLPL	M-HDL	0.025	0.014	0.079710401	0.146347166
	MHDLFC	M-HDL	0.019	0.016	0.230270494	0.307499446
	MHDLTG	M-HDL	0.032	0.017	0.060371337	0.118795211
	SHDLTG	S-HDL	0.066	0.017	0.000142621	0.002174971
	VLDLD	Lipoproteint particle size	0.023	0.017	0.16295363	0.25487619
	HDLD	Lipoproteint particle size	-0.037	0.016	0.02073047	0.048636872
	VLDLC	Cholesterol	0.020	0.016	0.214238117	0.303919189
	HDLC	Cholesterol	-0.009	0.016	0.56803118	0.587286474
	HDL2C	Cholesterol	-0.008	0.016	0.612285462	0.612285462
	SerumTG	Apolipoproteins & lipids	0.057	0.017	0.00095525	0.009711712
	VLDLTG	Apolipoproteins & lipids	0.048	0.017	0.004955744	0.033588931
	HDLTG	Apolipoproteins & lipids	0.037	0.017	0.032362832	0.068073544
	ApoA1	Apolipoproteins & lipids	0.009	0.016	0.562985302	0.587286474
	TotFA	Fatty acids & saturation	0.042	0.016	0.010671681	0.037985362
	MUFA	Fatty acids & saturation	0.073	0.016	3.23E-06	0.000141879
	Cit	Glycolysis	-0.024	0.012	0.037313	0.0597
	Ile	Amino acids	0.012	0.016	0.447466991	0.487419401
	Alb	Fluid balance	-0.051	0.016	0.000953246	0.009711712
	Gp	Inflammation	0.078	0.017	4.65E-06	0.000141879
	XLVLDLP	XL-VLDL	0.039	0.017	0.023172218	0.052352049
	XLVLDLL	XL-VLDL	0.038	0.017	0.025640696	0.055860088
	XLVLDLFC	XL-VLDL	0.031	0.017	0.072454056	0.138115545
	XSVLDLP	XS-VLDL	0.027	0.016	0.09258466	0.148622744
**Smoking**	XLVLDLC	XL-VLDL	0.020	0.023	0.3947495	0.609768398
	XLVLDLCE	XL-VLDL	0.022	0.024	0.359590751	0.60930655

	XLVLDLTG	XL-VLDL	0.028	0.023	0.219388674	0.431700295
	LVLDLP	L-VLDL	0.036	0.023	0.114453124	0.30683565
	LVLDLL	L-VLDL	0.036	0.023	0.12074431	0.30683565
	LVLDLPL	L-VLDL	0.034	0.023	0.135812501	0.30683565
	LVLDLC	L-VLDL	0.030	0.023	0.202887995	0.412538922
	LVLDLCE	L-VLDL	0.022	0.023	0.34728661	0.605270949
	LVLDLFC	L-VLDL	0.035	0.023	0.13160255	0.30683565
	LVLDLTG	L-VLDL	0.039	0.023	0.092133935	0.295798424
	MVLDLP	M-VLDL	0.036	0.023	0.122040853	0.30683565
	MVLDLL	M-VLDL	0.035	0.023	0.130960759	0.30683565
	MVLDLPL	M-VLDL	0.037	0.023	0.105722617	0.30683565
	MVLDLC	M-VLDL	0.021	0.023	0.370879969	0.609768398
	MVLDLCE	M-VLDL	0.004	0.023	0.854888343	0.861381563
	MVLDLFC	M-VLDL	0.038	0.023	0.099860678	0.304575068
	MVLDLTG	M-VLDL	0.039	0.023	0.086426779	0.295798424
	SVLDLP	S-VLDL	0.050	0.023	0.029005277	0.147972442
	SVLDLL	S-VLDL	0.047	0.022	0.036802819	0.172690149
	SVLDLPL	S-VLDL	0.060	0.023	0.00852165	0.064977579
	SVLDLC	S-VLDL	0.015	0.020	0.438544867	0.61641747
	SVLDLCE	S-VLDL	-0.004	0.019	0.835539047	0.861381563
	SVLDLFC	S-VLDL	0.046	0.021	0.025997319	0.147972442
	SVLDLTG	S-VLDL	0.060	0.023	0.010656373	0.072226531
	XSVLDLTG	XS-VLDL	0.103	0.023	8.89E-06	0.000180685
	XLHDLP	XL-HDL	-0.016	0.021	0.468458414	0.61641747
	XLHDLL	XL-HDL	-0.016	0.022	0.469904918	0.61641747
	XLHDLPL	XL-HDL	-0.018	0.021	0.388328028	0.609768398
	XLHDLC	XL-HDL	-0.012	0.022	0.595767746	0.68985552
	XLHDLCE	XL-HDL	-0.009	0.022	0.684371666	0.745476279
	XLHDLFC	XL-HDL	-0.018	0.022	0.417501035	0.609768398
	LHDLP	L-HDL	-0.012	0.021	0.5643485	0.675005068
	LHDLL	L-HDL	-0.013	0.021	0.545001584	0.664901933
	LHDLPL	L-HDL	-0.011	0.021	0.599382665	0.68985552
	LHDLC	L-HDL	-0.016	0.022	0.472362339	0.61641747
	LHDLCE	L-HDL	-0.015	0.022	0.492623015	0.61641747
	LHDLFC	L-HDL	-0.018	0.022	0.419840536	0.609768398
	MHDLL	M-HDL	0.013	0.019	0.495155017	0.61641747
	MHDLPL	M-HDL	0.025	0.019	0.184959346	0.389052418
	MHDLFC	M-HDL	0.031	0.022	0.151986484	0.331113411
	MHDLTG	M-HDL	0.023	0.023	0.321074405	0.576045255
	SHDLTG	S-HDL	0.078	0.023	0.000868207	0.008826773
	VLDLD	Lipoproteint particle size	0.006	0.022	0.798603773	0.839910865

	HDLD	Lipoproteint particle size	-0.037	0.022	0.088621035	0.295798424
	VLDLC	Cholesterol	0.018	0.022	0.406574174	0.609768398
	HDLC	Cholesterol	-0.006	0.021	0.764677437	0.818339011
	HDL2C	Cholesterol	-0.010	0.021	0.651877613	0.722991534
	SerumTG	Apolipoproteins & lipids	0.062	0.023	0.007777285	0.064977579
	VLDLTG	Apolipoproteins & lipids	0.046	0.023	0.044196152	0.192568948
	HDLTG	Apolipoproteins & lipids	0.042	0.023	0.072071806	0.293092012
	ApoA1	Apolipoproteins & lipids	0.010	0.021	0.61443565	0.694084716
	TotFA	Fatty acids & saturation	0.038	0.022	0.085705687	0.295798424
	MUFA	Fatty acids & saturation	0.084	0.021	6.77E-05	0.001033036
	Cit	Glycolysis	-0.079	0.016	4.44E-07	3.56E-06
	Ile	Amino acids	-0.004	0.021	0.861381563	0.861381563
	Alb	Fluid balance	-0.101	0.021	9.43E-07	2.87647E-05
	Gp	Inflammation	0.085	0.023	0.000178561	0.002178442
	XLVLDLP	XL-VLDL	0.027	0.023	0.242651821	0.462555034
	XLVLDLL	XL-VLDL	0.026	0.023	0.257671877	0.47630256
	XLVLDLFC	XL-VLDL	0.016	0.023	0.477809017	0.61641747
	XSVLDLP	XS-VLDL	0.048	0.022	0.029109333	0.147972442
**Vegetable intake** (medium serving/day)	XLVLDLC	XL-VLDL	-0.252	0.083	0.002404703	0.005617544
	XLVLDLCE	XL-VLDL	-0.243	0.083	0.003478377	0.006630656
	XLVLDLTG	XL-VLDL	-0.263	0.081	0.001255519	0.005617544
	LVLDLP	L-VLDL	-0.254	0.082	0.001870931	0.005617544
	LVLDLL	L-VLDL	-0.256	0.082	0.001745733	0.005617544
	LVLDLPL	L-VLDL	-0.255	0.081	0.001685083	0.005617544
	LVLDLC	L-VLDL	-0.244	0.083	0.003221025	0.006549417
	LVLDLCE	L-VLDL	-0.230	0.083	0.005419238	0.00972275
	LVLDLFC	L-VLDL	-0.274	0.082	0.000886881	0.005617544
	LVLDLTG	L-VLDL	-0.250	0.081	0.00212621	0.005617544
	MVLDLP	M-VLDL	-0.248	0.082	0.002578545	0.005617544
	MVLDLL	M-VLDL	-0.249	0.082	0.002463691	0.005617544
	MVLDLPL	M-VLDL	-0.253	0.082	0.002119663	0.005617544
	MVLDLC	M-VLDL	-0.253	0.083	0.002231439	0.005617544
	MVLDLCE	M-VLDL	-0.245	0.083	0.003083199	0.00648535
	MVLDLFC	M-VLDL	-0.249	0.082	0.002505259	0.005617544
	MVLDLTG	M-VLDL	-0.239	0.082	0.003417861	0.006630656
	SVLDLP	S-VLDL	-0.253	0.080	0.001592613	0.005617544
	SVLDLL	S-VLDL	-0.254	0.079	0.001371943	0.005617544
	SVLDLPL	S-VLDL	-0.242	0.080	0.002529971	0.005617544
	SVLDLC	S-VLDL	-0.228	0.069	0.00101827	0.005617544
	SVLDLCE	S-VLDL	-0.212	0.068	0.001821601	0.005617544
	SVLDLFC	S-VLDL	-0.232	0.073	0.001568817	0.005617544

	SVLDLTG	S-VLDL	-0.233	0.083	0.00484036	0.008947332
	XSVLDLTG	XS-VLDL	-0.220	0.082	0.007409156	0.012414449
	XLHDLP	XL-HDL	0.115	0.077	0.135138292	0.161635996
	XLHDLL	XL-HDL	0.112	0.077	0.143731626	0.168608254
	XLHDLPL	XL-HDL	0.157	0.075	0.03660531	0.046519248
	XLHDLC	XL-HDL	0.064	0.078	0.409143998	0.423013286
	XLHDLCE	XL-HDL	0.053	0.078	0.493860161	0.502091164
	XLHDLFC	XL-HDL	0.088	0.078	0.254845033	0.282646309
	LHDLP	L-HDL	0.202	0.076	0.007530076	0.012414449
	LHDLL	L-HDL	0.202	0.076	0.007784135	0.012495585
	LHDLPL	L-HDL	0.205	0.074	0.005980673	0.010423458
	LHDLC	L-HDL	0.198	0.077	0.010637338	0.01622194
	LHDLCE	L-HDL	0.201	0.077	0.009105068	0.014241261
	LHDLFC	L-HDL	0.184	0.078	0.01874629	0.027226754
	MHDLL	M-HDL	0.067	0.069	0.330498313	0.360007091
	MHDLPL	M-HDL	0.080	0.068	0.244885215	0.276629594
	MHDLFC	M-HDL	0.069	0.077	0.367879561	0.389666796
	MHDLTG	M-HDL	-0.187	0.080	0.019763672	0.028036836
	SHDLTG	S-HDL	-0.253	0.083	0.002165501	0.005617544
	VLDLD	Lipoproteint particle size	-0.182	0.080	0.022881969	0.03172273
	HDLD	Lipoproteint particle size	0.165	0.077	0.032412089	0.042981248
	VLDLC	Cholesterol	-0.260	0.078	0.000858214	0.005617544
	HDLC	Cholesterol	0.132	0.075	0.078262765	0.095480573
	HDL2C	Cholesterol	0.159	0.076	0.035608841	0.046215729
	SerumTG	Apolipoproteins & lipids	-0.262	0.082	0.001471669	0.005617544
	VLDLTG	Apolipoproteins & lipids	-0.252	0.082	0.002080494	0.005617544
	HDLTG	Apolipoproteins & lipids	-0.177	0.082	0.030497877	0.041341566
	ApoA1	Apolipoproteins & lipids	0.046	0.074	0.532347043	0.532347043
	TotFA	Fatty acids & saturation	-0.258	0.078	0.001006489	0.005617544
	MUFA	Fatty acids & saturation	-0.296	0.075	7.86E-05	0.004796545
	Cit	Glycolysis	0.003	0.050	0.956513	0.956513
	Ile	Amino acids	-0.154	0.076	0.041661674	0.051864533
	Alb	Fluid balance	-0.066	0.074	0.370502855	0.389666796
	Gp	Inflammation	-0.296	0.081	0.0002546	0.005617544
	XLVLDLP	XL-VLDL	-0.265	0.081	0.001120028	0.005617544
	XLVLDLL	XL-VLDL	-0.266	0.081	0.001069746	0.005617544
	XLVLDLFC	XL-VLDL	-0.280	0.082	0.000649495	0.005617544
	XSVLDLP	XS-VLDL	-0.196	0.078	0.011447179	0.017031168
**Fruit intake** (medium serving/day)	XLVLDLC	XL-VLDL	-0.030	0.066	0.645926867	0.846670521
	XLVLDLCE	XL-VLDL	-0.014	0.066	0.827224858	0.882683932
	XLVLDLTG	XL-VLDL	-0.074	0.064	0.25110439	0.645379972

	LVLDLP	L-VLDL	-0.065	0.064	0.314192897	0.645379972
	LVLDLL	L-VLDL	-0.064	0.064	0.317967277	0.645379972
	LVLDLPL	L-VLDL	-0.072	0.064	0.25895411	0.645379972
	LVLDLC	L-VLDL	-0.035	0.065	0.591251526	0.841744825
	LVLDLCE	L-VLDL	-0.013	0.065	0.839273247	0.882683932
	LVLDLFC	L-VLDL	-0.069	0.065	0.289491501	0.645379972
	LVLDLTG	L-VLDL	-0.067	0.064	0.296212797	0.645379972
	MVLDLP	M-VLDL	-0.056	0.065	0.384858523	0.690481468
	MVLDLL	M-VLDL	-0.055	0.065	0.399062234	0.695508464
	MVLDLPL	M-VLDL	-0.068	0.065	0.294703571	0.645379972
	MVLDLC	M-VLDL	-0.023	0.065	0.730302437	0.882683932
	MVLDLCE	M-VLDL	0.011	0.065	0.869464487	0.883955562
	MVLDLFC	M-VLDL	-0.058	0.065	0.372025059	0.687682685
	MVLDLTG	M-VLDL	-0.063	0.064	0.327979986	0.645379972
	SVLDLP	S-VLDL	-0.020	0.063	0.755167731	0.882683932
	SVLDLL	S-VLDL	-0.011	0.063	0.860122263	0.883955562
	SVLDLPL	S-VLDL	-0.034	0.063	0.593361106	0.841744825
	SVLDLC	S-VLDL	0.071	0.055	0.196554503	0.645379972
	SVLDLCE	S-VLDL	0.091	0.054	0.090949408	0.504355806
	SVLDLFC	S-VLDL	0.014	0.058	0.80674644	0.882683932
	SVLDLTG	S-VLDL	-0.053	0.065	0.419492521	0.710806771
	XSVLDLTG	XS-VLDL	-0.029	0.065	0.655139438	0.846670521
	XLHDLP	XL-HDL	-0.026	0.061	0.666232541	0.846670521
	XLHDLL	XL-HDL	-0.027	0.061	0.661022393	0.846670521
	XLHDLPL	XL-HDL	-0.035	0.059	0.55844498	0.830857166
	XLHDLC	XL-HDL	-0.015	0.062	0.811536726	0.882683932
	XLHDLCE	XL-HDL	-0.002	0.062	0.974269045	0.974269045
	XLHDLFC	XL-HDL	-0.045	0.061	0.460566673	0.739330713
	LHDLP	L-HDL	-0.039	0.060	0.516031968	0.807126925
	LHDLL	L-HDL	-0.036	0.060	0.543955702	0.829532445
	LHDLPL	L-HDL	-0.059	0.059	0.317466802	0.645379972
	LHDLC	L-HDL	-0.018	0.061	0.773214233	0.882683932
	LHDLCE	L-HDL	-0.019	0.061	0.753097735	0.882683932
	LHDLFC	L-HDL	-0.013	0.062	0.83781385	0.882683932
	MHDLL	M-HDL	-0.158	0.054	0.003602343	0.072713257
	MHDLPL	M-HDL	-0.163	0.054	0.002494569	0.072713257
	MHDLFC	M-HDL	-0.113	0.061	0.063723373	0.504355806
	MHDLTG	M-HDL	-0.088	0.063	0.162525767	0.645379972
	SHDLTG	S-HDL	-0.083	0.065	0.202256933	0.645379972
	VLDLD	Lipoproteint particle size	-0.074	0.063	0.243765494	0.645379972
	HDLD	Lipoproteint particle size	0.047	0.061	0.438421228	0.722802565

	VLDLC	Cholesterol	0.016	0.062	0.800821186	0.882683932
	HDLC	Cholesterol	-0.082	0.059	0.166715301	0.645379972
	HDL2C	Cholesterol	-0.087	0.060	0.145309045	0.645379972
	SerumTG	Apolipoproteins & lipids	-0.074	0.065	0.252297397	0.645379972
	VLDLTG	Apolipoproteins & lipids	-0.065	0.065	0.318219492	0.645379972
	HDLTG	Apolipoproteins & lipids	-0.072	0.065	0.266821019	0.645379972
	ApoA1	Apolipoproteins & lipids	-0.101	0.059	0.085196141	0.504355806
	TotFA	Fatty acids & saturation	-0.120	0.062	0.051825928	0.504355806
	MUFA	Fatty acids & saturation	-0.166	0.059	0.00519113	0.072713257
	Cit	Glycolysis	-0.160	0.041	8.45E-05	0.000225
	Ile	Amino acids	-0.103	0.060	0.084249864	0.504355806
	Alb	Fluid balance	-0.103	0.058	0.079191315	0.504355806
	Gp	Inflammation	-0.180	0.064	0.004726837	0.072713257
	XLVLDLP	XL-VLDL	-0.071	0.064	0.270165695	0.645379972
	XLVLDLL	XL-VLDL	-0.070	0.064	0.279204126	0.645379972
	XLVLDLFC	XL-VLDL	-0.061	0.065	0.344633974	0.656958513
	XSVLDLP	XS-VLDL	0.029	0.061	0.635753746	0.846670521
**Fish intake** (medium serving/day)	XLVLDLC	XL-VLDL	-0.139	0.088	0.116510707	0.302218984
	XLVLDLCE	XL-VLDL	-0.138	0.089	0.119714302	0.302218984
	XLVLDLTG	XL-VLDL	-0.170	0.087	0.049447931	0.302218984
	LVLDLP	L-VLDL	-0.150	0.087	0.085671249	0.302218984
	LVLDLL	L-VLDL	-0.149	0.087	0.086892646	0.302218984
	LVLDLPL	L-VLDL	-0.140	0.087	0.107156808	0.302218984
	LVLDLC	L-VLDL	-0.141	0.088	0.10836447	0.302218984
	LVLDLCE	L-VLDL	-0.141	0.088	0.10930441	0.302218984
	LVLDLFC	L-VLDL	-0.146	0.088	0.095308903	0.302218984
	LVLDLTG	L-VLDL	-0.151	0.087	0.082176811	0.302218984
	MVLDLP	M-VLDL	-0.116	0.088	0.184545075	0.335207374
	MVLDLL	M-VLDL	-0.116	0.088	0.186836897	0.335207374
	MVLDLPL	M-VLDL	-0.127	0.088	0.148462066	0.314747703
	MVLDLC	M-VLDL	-0.099	0.088	0.261515668	0.419801466
	MVLDLCE	M-VLDL	-0.081	0.088	0.36068551	0.500041275
	MVLDLFC	M-VLDL	-0.114	0.088	0.192929	0.336247685
	MVLDLTG	M-VLDL	-0.116	0.087	0.181766705	0.335207374
	SVLDLP	S-VLDL	-0.053	0.086	0.535847961	0.68097345
	SVLDLL	S-VLDL	-0.047	0.084	0.581439589	0.723832958
	SVLDLPL	S-VLDL	-0.080	0.085	0.34753752	0.500041275
	SVLDLC	S-VLDL	0.024	0.074	0.74812	0.853065812
	SVLDLCE	S-VLDL	0.054	0.073	0.459109027	0.595864907
	SVLDLFC	S-VLDL	-0.036	0.078	0.644894747	0.771344697
	SVLDLTG	S-VLDL	-0.076	0.088	0.385106927	0.522033834

	XSVLDLTG	XS-VLDL	0.014	0.087	0.870648489	0.949147891
	XLHDLP	XL-HDL	0.132	0.082	0.105757311	0.302218984
	XLHDLL	XL-HDL	0.131	0.082	0.108001016	0.302218984
	XLHDLPL	XL-HDL	0.129	0.080	0.105700555	0.302218984
	XLHDLC	XL-HDL	0.126	0.083	0.129711683	0.302218984
	XLHDLCE	XL-HDL	0.126	0.083	0.128624303	0.302218984
	XLHDLFC	XL-HDL	0.119	0.083	0.149634154	0.314747703
	LHDLP	L-HDL	0.143	0.081	0.076549609	0.302218984
	LHDLL	L-HDL	0.140	0.081	0.082967189	0.302218984
	LHDLPL	L-HDL	0.128	0.079	0.106802314	0.302218984
	LHDLC	L-HDL	0.140	0.082	0.089419183	0.302218984
	LHDLCE	L-HDL	0.149	0.082	0.069802657	0.302218984
	LHDLFC	L-HDL	0.110	0.083	0.184884704	0.335207374
	MHDLL	M-HDL	-0.001	0.074	0.988239809	0.98978406
	MHDLPL	M-HDL	0.001	0.073	0.98978406	0.98978406
	MHDLFC	M-HDL	0.068	0.082	0.408088933	0.541161411
	MHDLTG	M-HDL	-0.098	0.086	0.251403135	0.414475439
	SHDLTG	S-HDL	-0.081	0.088	0.359497935	0.500041275
	VLDLD	Lipoproteint particle size	-0.159	0.085	0.062415658	0.302218984
	HDLD	Lipoproteint particle size	0.123	0.082	0.133769058	0.302218984
	VLDLC	Cholesterol	-0.039	0.083	0.640135108	0.771344697
	HDLC	Cholesterol	0.137	0.080	0.086055405	0.302218984
	HDL2C	Cholesterol	0.096	0.081	0.234921996	0.39806227
	SerumTG	Apolipoproteins & lipids	-0.094	0.088	0.285970614	0.440335428
	VLDLTG	Apolipoproteins & lipids	-0.120	0.087	0.168346593	0.335207374
	HDLTG	Apolipoproteins & lipids	0.006	0.087	0.947730523	0.979856981
	ApoA1	Apolipoproteins & lipids	0.084	0.079	0.288744543	0.440335428
	TotFA	Fatty acids & saturation	-0.007	0.084	0.928758076	0.976797286
	MUFA	Fatty acids & saturation	-0.027	0.080	0.737460397	0.853065812
	Cit	Glycolysis	-0.130	0.062	0.035479	0.094611
	Ile	Amino acids	0.025	0.081	0.755173014	0.853065812
	Alb	Fluid balance	0.013	0.079	0.871348883	0.949147891
	Gp	Inflammation	0.009	0.086	0.915859152	0.976797286
	XLVLDLP	XL-VLDL	-0.164	0.087	0.058795995	0.302218984
	XLVLDLL	XL-VLDL	-0.162	0.087	0.061372494	0.302218984
	XLVLDLFC	XL-VLDL	-0.145	0.088	0.097624509	0.302218984
	XSVLDLP	XS-VLDL	0.082	0.083	0.319205227	0.474915095

Subjects who had data on genetics, lifestyle factors and NMR metabolite measurements (n = 3836). Pooled linear regression analyses adjusted for age, sex and study site. Abbreviations: GRS = genetic risk score; LRS = lifestyle risk score; SE = standard error; FDR = false discovery rate.

Supplementary Table S11. Associations between GRS, LRS and metabolites (late AMD)

Determinant	Outcome	Outcome type	Estimate	SE	P-value	FDR-adjusted P-value
GRS total	Cit	Glycolysis	0.038	0.011	0.000541	0.001442
	His	Amino Acids	-0.039	0.015	0.006734	0.010774

	Leu	Amino Acids	0.000	0.012	0.982767	0.982767
	Val	Amino Acids	-0.009	0.013	0.525417	0.600477
	Phe	Amino Acids	0.044	0.010	8.12E-06	6.49E-05
	Tyr	Amino Acids	-0.023	0.014	0.107737	0.143649
	AcAce	Ketone bodies	0.052	0.015	0.000526	0.001442
	bOHBut	Ketone bodies	0.043	0.014	0.002169	0.004338
GRS complement	Cit	Glycolysis	0.027	0.011	0.015141	0.027325
	His	Amino Acids	-0.045	0.015	0.00209	0.016718
	Leu	Amino Acids	-0.015	0.012	0.222757	0.222757
	Val	Amino Acids	-0.026	0.014	0.055718	0.071233
	Phe	Amino Acids	0.028	0.010	0.005328	0.02131
	Tyr	Amino Acids	-0.034	0.014	0.017078	0.027325
	AcAce	Ketone bodies	0.028	0.015	0.062329	0.071233
	bOHBut	Ketone bodies	0.038	0.014	0.008308	0.022154
GRS lipid	Cit	Glycolysis	-0.011	0.011	0.335904	0.537446
	His	Amino Acids	0.006	0.015	0.673106	0.769265
	Leu	Amino Acids	0.010	0.012	0.417894	0.557191
	Val	Amino Acids	0.021	0.014	0.130908	0.36543
	Phe	Amino Acids	0.010	0.010	0.33213	0.537446
	Tyr	Amino Acids	0.028	0.014	0.053268	0.36543
	AcAce	Ketone bodies	-0.004	0.015	0.795674	0.795674
	bOHBut	Ketone bodies	-0.022	0.014	0.137036	0.36543
GRS ECM	Cit	Glycolysis	-0.002	0.011	0.819322	0.936368
	His	Amino Acids	0.012	0.014	0.393915	0.52749
	Leu	Amino Acids	0.019	0.012	0.120707	0.500194
	Val	Amino Acids	0.012	0.013	0.355156	0.52749
	Phe	Amino Acids	0.013	0.010	0.167193	0.500194
	Tyr	Amino Acids	0.018	0.014	0.187573	0.500194
	AcAce	Ketone bodies	0.001	0.015	0.951207	0.951207
	bOHBut	Ketone bodies	0.012	0.014	0.395618	0.52749
ARMS2/HTRA1	Cit	Glycolysis	0.039	0.011	0.000222	0.001281
	His	Amino Acids	-0.013	0.014	0.363838	0.582141
	Leu	Amino Acids	0.006	0.012	0.620904	0.704758
	Val	Amino Acids	0.005	0.013	0.704758	0.704758
	Phe	Amino Acids	0.030	0.010	0.001727	0.004605
	Tyr	Amino Acids	-0.010	0.014	0.4845	0.646
	AcAce	Ketone bodies	0.052	0.015	0.00032	0.001281
	bOHBut	Ketone bodies	0.029	0.014	0.036286	0.072573
LRS	Cit	Glycolysis	-0.024	0.012	0.037313	0.0597
	His	Amino Acids	-0.041	0.015	0.00746	0.01492
	Leu	Amino Acids	-0.010	0.013	0.442777	0.442777

	Val	Amino Acids	-0.055	0.014	0.00013	0.001042
	Phe	Amino Acids	0.015	0.011	0.153677	0.175631
	Tyr	Amino Acids	-0.042	0.015	0.005218	0.013914
	AcAce	Ketone bodies	0.023	0.016	0.137196	0.175631
	bOHBut	Ketone bodies	0.044	0.015	0.002902	0.011609
Smoking	Cit	Glycolysis	-0.079	0.016	4.44E-07	3.56E-06
	His	Amino Acids	-0.054	0.021	0.010245	0.02732
	Leu	Amino Acids	-0.019	0.018	0.295521	0.409118
	Val	Amino Acids	-0.054	0.019	0.005691	0.022765
	Phe	Amino Acids	0.013	0.014	0.357978	0.409118
	Tyr	Amino Acids	-0.029	0.021	0.166603	0.333207
	AcAce	Ketone bodies	0.008	0.022	0.722521	0.722521
	bOHBut	Ketone bodies	0.020	0.021	0.326309	0.409118
Vegetable intake (medium serving/day)	Cit	Glycolysis	0.003	0.050	0.956513	0.956513
	His	Amino Acids	0.097	0.067	0.143793	0.287587
	Leu	Amino Acids	-0.008	0.058	0.884428	0.956513
	Val	Amino Acids	0.154	0.062	0.013282	0.053127
	Phe	Amino Acids	0.068	0.046	0.13785	0.287587
	Tyr	Amino Acids	0.073	0.066	0.267509	0.356679
	AcAce	Ketone bodies	-0.083	0.068	0.222195	0.355512
	bOHBut	Ketone bodies	-0.216	0.064	0.000769	0.00615
Fruit intake (medium serving/day)	Cit	Glycolysis	-0.160	0.041	8.45E-05	0.000225
	His	Amino Acids	0.095	0.054	0.076634	0.102178
	Leu	Amino Acids	-0.069	0.047	0.14623	0.16712
	Val	Amino Acids	0.030	0.050	0.551899	0.551899
	Phe	Amino Acids	-0.150	0.037	5.19E-05	0.000208
	Tyr	Amino Acids	0.135	0.053	0.011443	0.018309
	AcAce	Ketone bodies	-0.146	0.055	0.007679	0.015358
	bOHBut	Ketone bodies	-0.260	0.052	6.09E-07	4.87E-06
Fish intake (medium serving/day)	Cit	Glycolysis	-0.130	0.062	0.035479	0.094611
	His	Amino Acids	0.037	0.082	0.654615	0.872819
	Leu	Amino Acids	0.121	0.072	0.091339	0.178369
	Val	Amino Acids	0.295	0.076	0.000112	0.0009
	Phe	Amino Acids	0.089	0.056	0.111481	0.178369
	Tyr	Amino Acids	0.250	0.081	0.002035	0.008142
	AcAce	Ketone bodies	-0.023	0.083	0.779948	0.891369
	bOHBut	Ketone bodies	0.001	0.079	0.986617	0.986617

Subjects who had data on genetics, lifestyle factors and NMR metabolite measurements (n = 3836). Pooled linear regression analyses adjusted for age, sex and study site. Abbreviations: GRS = genetic risk score; LRS = lifestyle risk score; SE = standard error; FDR = false discovery rate.

Table S12. Association of genetic, lifestyle and metabolite risk scores with late AMD

	**Late AMD** (n = 704)

**Risk scores**	**OR**	**95% CI**	*P-value*
Genetics	2.1	(1.9 – 2.3)	<2e-16
Lifestyle	1.3	(1.0 – 1.8)	0.05
Metabolites	1.5	(1.4 – 1.7)	<2e-16

Multivariable generalized linear mixed model with study site as a random factor. After matching for age and sex. Reference = no AMD. Abbreviations: OR = odds ratio; CI = confidence interval.
